# Tumor biomarkers and efficacy in patients treated with trastuzumab emtansine + pertuzumab versus standard of care in HER2-positive early breast cancer: an open-label, phase III study (KRISTINE)

**DOI:** 10.1186/s13058-022-01587-z

**Published:** 2023-01-11

**Authors:** Sanne L. de Haas, Dennis J. Slamon, Miguel Martin, Michael F. Press, Gail D. Lewis, Chiara Lambertini, Aleix Prat, Vanesa Lopez-Valverde, Thomas Boulet, Sara A. Hurvitz

**Affiliations:** 1 Oncology Biomarker Development, F. Hoffmann-La Roche Ltd., Grenzacherstrasse 124, 4070 Basel, Switzerland; 2 Division of Hematology/Oncology, David Geffen School of Medicine, University of California Los Angeles, Los Angeles, CA USA; 3 Medical Oncology Service, Hospital Gregorio Marañón, Universidad Complutense, CIBERONC, Madrid, Spain; 4 Department of Pathology, Keck School of Medicine, USC/Norris Comprehensive Cancer Center, Los Angeles, CA USA; 5 Discovery Oncology, Genentech, Inc., South San Francisco, CA USA; 6 Translational Genomics and Targeted Therapies in Solid Tumors, IDIBAPS, Barcelona, Spain; 7 Medical Oncology, Hospital Clinic of Barcelona, Barcelona, Spain; 8 Department of Medicine, University of Barcelona, Barcelona, Spain; 9 Pharmaceuticals Division, F. Hoffmann-La Roche Ltd., Basel, Switzerland; 10 Product Development Biometrics Biostatistics, F. Hoffmann-La Roche Ltd., Basel, Switzerland; 11 Division of Hematology-Oncology, David Geffen School of Medicine, Jonsson Comprehensive Cancer Center, University of California Los Angeles, Los Angeles, CA USA

**Keywords:** Tumor biomarkers, Trastuzumab emtansine, Pertuzumab, HER2-positive breast cancer, KRISTINE

## Abstract

**Background:**

KRISTINE is an open-label, phase III study of trastuzumab emtansine + pertuzumab (T-DM1 + P) versus docetaxel + carboplatin + trastuzumab + pertuzumab (TCH + P) in patients with HER2-positive, stage II–III breast cancer. We investigated the association of biomarkers with clinical outcomes in KRISTINE.

**Methods:**

Patients were randomized to receive neoadjuvant T-DM1 + P or TCH + P and assessed for pathologic complete response (pCR; ypT0/is, ypN0). HER2 status (per central assessment), hormone receptor status, *PIK3CA* mutation status, HER2/HER3 mRNA levels, tumor-infiltrating lymphocyte levels, PD-L1 status, and NanoString data were analyzed. pCR rates by treatment arm were compared across biomarker subgroups. Analyses were descriptive.

**Results:**

Biomarker analyses included data from all 444 patients (T-DM1 + P, *n* = 223; TCH + P, *n* = 221) enrolled in KRISTINE. Biomarker distribution was balanced across treatment arms. All subgroups with higher HER2 amplification/expression and immune marker levels showed numerically higher pCR rates in both arms. Mutated versus non-mutated *PIK3CA* tumors were associated with numerically lower pCR rates in the T-DM1 + P arm but not in the TCH + P arm. In a multivariate analysis, Prediction Analysis of Microarray with the 50-gene classifier (PAM50) HER2-enriched subtype, HER2 gene ratio ≥ 4, and PD-L1-positive status positively influenced the pCR rate. Biomarkers associated with lower pCR rates (e.g., low HER2 levels, positive hormone receptor status, mutated *PIK3CA*) were more likely to co-occur. Dynamic on-treatment biomarker changes were observed. Differences in the treatment effects for T-DM1 + P versus TCH + P were similar to those observed in the intent-to-treat population for the majority of the biomarker subgroups.

**Conclusions:**

Although our biomarker analysis did not identify a subgroup of patients that benefited from neoadjuvant T-DM1 + P versus TCH + P, the data revealed that patients with higher HER2 amplification/expression and immune marker levels had improved response irrespective of treatment arm. These analyses confirm the role of HER2 tumor biology and the immune microenvironment in influencing pCR in the neoadjuvant setting and reaffirm the molecular diversity of HER2-positive breast cancer.

*Trial Registration*: ClinicalTrials.gov NCT02131064. Registered 06 May 2014.

**Supplementary Information:**

The online version contains supplementary material available at 10.1186/s13058-022-01587-z.

## Background

The antibody–drug conjugate (ADC) trastuzumab emtansine (T-DM1) is composed of the anti-human epidermal growth factor receptor 2 (HER2) antibody trastuzumab linked to the cytotoxic agent DM1 through a stable, uncleavable linker [1]. T-DM1 selectively delivers DM1 to HER2-positive tumor cells and retains the mechanisms of action of trastuzumab [2]. Based on results from a randomized phase III study (EMILIA) [3], T-DM1 was approved by the US Food and Drug Administration (FDA) in 2013 for the treatment of patients with HER2-positive metastatic breast cancer (mBC) who previously received trastuzumab and a taxane. T-DM1 is also FDA-approved as an adjuvant treatment in patients with HER2-positive early breast cancer (eBC) with residual invasive disease after neoadjuvant taxane and trastuzumab-based treatment, based on findings from the KATHERINE study [4, 5].

Pertuzumab is an anti-HER2 antibody that differs from trastuzumab by blocking HER2 dimerization with other HER family members, thereby inhibiting ligand-dependent HER2 signaling [6]. In combination with trastuzumab and docetaxel, pertuzumab is FDA-approved for the first-line treatment of HER2-positive mBC [7] and in the neoadjuvant setting for patients with HER2-positive, locally advanced, inflammatory, or eBC based on demonstration of improvements in pathologic complete response (pCR) and invasive disease-free survival (iDFS) rates versus trastuzumab and chemotherapy [5, 8]. In the phase III MARIANNE study, first-line treatment with T-DM1 alone or in combination with pertuzumab (T-DM1 + P) was associated with non-inferior efficacy but better tolerability than trastuzumab plus taxane in patients with HER2-positive mBC [9]. T-DM1 + P was also assessed in patients with HER2-positive stage II/III breast cancer in KRISTINE, a phase III study that compared neoadjuvant T-DM1 + P with docetaxel, carboplatin, and trastuzumab plus pertuzumab (TCH + P) [10, 11]. As reported previously, the pCR rate was higher in the TCH + P arm versus T-DM1 + P (55.7% vs. 44.4%; absolute difference − 11.3 percentage points, 95% confidence interval [CI] – 20.5 to – 2.0; *P* = 0.016) [10]. Event-free survival (EFS) at 3 years numerically favored TCH + P, with a higher number of locoregional progression events before surgery in patients treated with T-DM1 + P; iDFS after surgery was similar between arms [11].

Biomarker analyses could potentially elucidate patient subgroups with better prognosis or with a higher likelihood of clinical benefit from treatment with specific HER2-targeted regimens (e.g., T-DM1 + P vs. TCH + P) in the adjuvant/neoadjuvant setting [12]. Exploratory biomarker analyses from previous studies of HER2-targeted therapy have shown associations between the levels of HER2 amplification and/or expression and outcomes in both mBC [13–15] and eBC [16, 17]. Consistent with these data, the HER2-enriched (HER2-E) intrinsic breast cancer subtype has been associated with better responses [18–21] and favorable long-term outcomes in patients treated with neoadjuvant HER2-targeted therapy [18, 21, 22]. In addition, increased HER2 heterogeneity has been associated with reduced benefit from T-DM1 [15]. Preclinical and clinical evidence suggests that mutated (vs. non-mutated) phosphatidylinositol-4,5-bisphosphate 3-kinase catalytic subunit alpha (*PIK3CA*) confers resistance to HER2-targeted therapies [17, 23]. Markers indicating an activated immune microenvironment, such as higher tumor-infiltrating lymphocytes (TILs) [24–26] or higher expression of immune gene signatures, are associated with higher pCR rates [19, 27] and better long-term outcomes in HER2-positive BC [23, 28]. Other biomarkers have also been hypothesized to influence outcomes, such as the anti-apoptotic marker bcl-2 [29] and expression of the MDR1/ABCB1 drug efflux pump, for which upregulation has been associated with resistance to T-DM1 and other therapeutics in breast cancer [30–32]. Here, we report the results obtained from exploratory analyses designed to examine a variety of biomarkers and their association with clinical outcomes in patients who participated in the KRISTINE study.

## Methods

### Study design and patients

The KRISTINE study design and results from primary and secondary analyses have been reported elsewhere [10, 11]. Briefly, KRISTINE (ClinicalTrials.gov #NCT02131064) was a randomized, multicenter, open-label, phase III study of treatment-naïve patients with HER2-positive, operable, stage II/III breast cancer. All tumors scored as HER2-positive according to local testing were confirmed by central testing. Patients were randomized (1:1) to receive six cycles (administered every 3 weeks) of neoadjuvant T-DM1 + P (T-DM1: 3.6 mg/kg; P: 840 mg loading dose, then 420 mg maintenance doses) or TCH + P (docetaxel: 75 mg/m^2^; carboplatin: area under the concentration–time curve 6 mg/mL × min; trastuzumab: 8 mg/kg loading dose, then 6 mg/kg maintenance doses; P: 840 mg loading dose, then 420 mg maintenance doses). All drugs were administered intravenously. HER2-targeted therapy continued within 9 weeks after surgery for a maximum of 18 total cycles in the neoadjuvant/adjuvant settings. At the discretion of the treating physician, patients treated with neoadjuvant T-DM1 + P could initiate adjuvant chemotherapy.

The primary efficacy endpoint was locally determined pCR (ypT0/is, ypN0), assessed using samples taken at surgery following the completion of neoadjuvant therapy [10]. Secondary endpoints included EFS (defined as the time from randomization to disease progression, disease recurrence, or death from any cause), iDFS, and overall survival. Exploratory biomarker analyses (both predefined and post hoc) were conducted to evaluate potential associations between tissue biomarkers and pCR in each treatment arm. The association of selected biomarkers of interest with EFS was also assessed, but using data pooled from both treatment arms due to the low event rate. The iDFS event rate was too low to enable any assessment of association with tissue biomarkers.

The KRISTINE study protocol, which included a description of preplanned exploratory biomarker analyses, received ethics approval from the institutional review board or ethics committee at each study site, and all patients provided written informed consent. The study was conducted in accordance with the International Conference on Harmonization Good Clinical Practice guidelines and the Declaration of Helsinki.

### Biomarker assessments

Biomarker analyses were conducted on formalin-fixed paraffin-embedded tumor tissue collected at baseline for all markers (Additional file 1: Table S1) and at cycle 2 and surgery for selected markers. HER2 status used for study eligibility was centrally assessed at baseline at Targos Molecular Pathology GmbH (Kassel, Germany). HER2 positivity was defined as immunohistochemistry 3 + (IHC3+) (using PATHWAY anti-HER2/neu [4B5] assay, Ventana Medical Systems, Roche Diagnostics) and/or HER2/CEP17 gene ratio ≥ 2 by in situ hybridization (using ISH; INFORM HER2 Dual ISH assay, Ventana Medical Systems, Roche Diagnostics). As part of the central HER2 assessment, the percentage and intensity of HER2 staining was assessed manually by central laboratory pathologists and categorized as focal (< 30% of cells positive for HER2), heterogeneous (30–79%), or homogeneous (≥ 80%) in the subgroup of patients with HER2 IHC2+ or IHC3+ staining, as previously described [15] (Additional file 1: Fig. S1). HER2 H-score was calculated as ((%IHC3+) × 3) + ((%IHC2+) × 2) + (%IHC1+) [15, 33]. HER2 and HER3 messenger RNA (mRNA) expression were measured at baseline using quantitative reverse transcriptase polymerase chain reaction (qRT-PCR) using the cobas® z 480 analyzer (Roche Diagnostics). *PIK3CA* mutation status was determined at baseline using the cobas® *PIK3CA* Mutation Test on the cobas® z 480 analyzer (both Roche Diagnostics).

Expression of additional predefined single genes and signatures of interest were analyzed using a custom 800-gene codeset on the nCounter® platform (NanoString Technologies) from samples collected at baseline, cycle 2, and surgery. Genes of interest included HER2, programmed death ligand 1 (PD-L1), CD8, predefined immune signatures including 3-gene (PD-L1/IFNG/CXCL9), 5-gene (PD-L1/granzyme B/CD8/IFNG/CXCL9), T effector (Teff) signature (CD8/granzyme A/granzyme B/perforin/IFNG), chemokine signaling (ThCytokines signature, i.e., CXCL9/CXCL10/CXCL11), and checkpoint inhibitor signatures (PD-L1/PD-L2/IDO), as well as other markers of interest (complete listing available in Additional file 1: Table S2) based on knowledge of the immune signatures and TILs associated with pCR in HER2-positive eBC [17, 19].

The Prediction Analysis of Microarray with the 50-gene classifier (PAM50) subtype prediction [34] was carried out using the random-forest-based classifier [35] and examined HER2-E, luminal A, luminal B, and basal-like as the major intrinsic breast cancer subtypes.

Expression of PD-L1 by IHC was assessed post hoc (Ventana SP142 assay, Roche Diagnostics) from samples collected at baseline, cycle 2, and surgery. PD-L1-positive disease was defined as tumor-infiltrating immune cell (IC) expression ≥ 1% of the tumor area (IC score 1, 2, or 3), while PD-L1-negative disease was defined as PD-L1 expression on IC < 1% of the tumor area (IC score 0) [36]. Stromal TILs were assessed post hoc using hematoxylin and eosin images from tumor samples according to the International Immuno-oncology Biomarker Working Group on Breast Cancer guidelines [25] from samples collected at baseline, cycle 2, and surgery. A predefined cutoff was used to define high- (> 10%) and low- (≤ 10%) infiltrated tumors.

Local hormone receptor status was assessed as a stratification factor, and centrally assessed hormone receptor status was used for the biomarker analyses. Hormone receptor-positive disease was defined as estrogen and/or progesterone receptor staining of ≥ 1% (Dako Omnis IHC assay, Agilent Technologies).

FcγR IIa (rs1801274), FcγR IIIa (s396991), ABCB1 polymorphisms (RS1045642), and ABCG2 polymorphisms (RS2231142) were analyzed based on whole blood samples collected at baseline or during the study. Polymorphisms were assessed using genotyping assays from ThermoFisher and TaqMan probe chemistry.

All biomarkers were analyzed for their correlation with pCR. In addition, specific biomarkers (HER2 IHC subgroups, *PIK3CA* mutation status, and PD-L1 status) were measured from baseline samples and evaluated for their correlation with EFS in a pooled analysis of both treatment arms. These biomarkers were selected for EFS analyses based on their potential as targets of possible combination therapies and the robustness of the assays.

### Statistical methods

pCR rates by treatment arm were assessed across biomarker subgroups for HER2 IHC (IHC1+, IHC2+, or IHC3+), HER2/CEP17 gene ratio (≥ 2 to < 4 or ≥ 4), HER2 and HER3 mRNA expression (≤ median or > median), HER2 staining pattern (focal, heterogeneous, or homogeneous), *PIK3CA* mutation status (mutated or non-mutated), intrinsic breast cancer subtype (HER2-E, luminal A, luminal B, or basal-like), PD-L1 expression (IC 0 or IC 1, 2, or 3), stromal TILs (≤ 10% or > 10%), single genes of interest, and immune genes/signatures (≤ median or > median).

A multivariate analysis was carried out to identify biomarkers most prognostic for pCR, regardless of treatment. Univariate analysis based on a logistic model was conducted for all biomarkers assessed. Biomarkers showing at least a moderate potential association with pCR (< 0.15 significance level) were included in a multivariate logistic model in which backwards selection was applied. The final analysis model included randomized treatment plus the set of biomarker covariates selected based on the previous step.

An unstratified Cox proportional hazards model was used to estimate hazard ratios (HRs) between biomarker subgroups (preplanned: HER2 IHC subgroups [3+ versus 2+], PD-L1 IHC [IC 1/2/3 versus IC 0], and PIK3CA [mutated versus non-mutated]; post hoc: PAM50 subtype [HER2-E versus non-HER2-E]) and corresponding 95% CIs for the EFS endpoint. The Kaplan–Meier approach was used to estimate 3-year EFS rates and corresponding 95% CIs.

Changes in PAM50 subtype, PD-L1 IHC status, percentage of stromal TILs, and HER2 mRNA levels (NanoString) from baseline to cycle 2 and surgery were assessed by treatment arm.

As these were exploratory analyses, all gene expression levels, HRs, odds ratios, and 95% CIs should be considered descriptive and are included for illustrative purposes only. The clinical cutoff dates for the biomarker analyses were the same as those used for the primary and secondary endpoints: December 3, 2015, for the pCR analysis and May 29, 2018, for the final EFS analysis.

## Results

### Patients

Data from all 444 patients (T-DM1 + P, *n* = 223; TCH + P, *n* = 221) enrolled in KRISTINE were included in the biomarker analyses (Additional file 1: Fig. S2). Baseline characteristics for this population have been reported [10]. HER2-related biomarkers (HER2 by IHC, including HER2-variable IHC staining; HER2 by ISH, including gene ratio; and HER2 and HER3 mRNA by qRT-PCR), *PIK3CA* mutation status, PAM50 subtype, and PD-L1 IHC status at baseline were generally balanced across the two treatment arms, although the prevalence of the HER2-E subtype was slightly higher in the TCH + P arm (Table 1). For patients with a central hormonal receptor status assessment available (*n* = 424), concordance between local and central assessment was achieved in 368 patients (86.8%; 235/424 [55.4%] for hormone receptor-positive disease and 133/424 [31.4%] for hormone receptor-negative disease). Post-surgical chemotherapy was administered to 50/204 (24.5%) patients in the T-DM1 + P arm (41/124 [33.1%] patients with residual disease and 9/80 [11.3%] patients with pCR) and was not administered to any patients in the TCH + P arm.

**Table 1:** Prevalence of key biomarkers at baseline

Biomarker	TCH + P *N* = 221	T-DM1 + P *N* = 223
HER2, *n* (%)	221 (100.0)	223 (100.0)
ISH+/IHC3+	184 (83.3)	185 (83.0)
ISH+/IHC2+	25 (11.3)	26 (11.7)
ISH+/IHC0 or 1+	2 (0.9)	0 (0)
ISH−/IHC3+	1 (0.5)	2 (0.9)
ISH−/IHC2+	0 (0)	1 (0.4)^a^
ISH unknown/IHC3+	9 (4.1)	8 (3.6)
ISH unknown/IHC2+	0 (0)	1 (0.4)^a^
HER2/CEP17 gene ratio, *n* (%)	211 (95.5)	210 (94.2)
≥ 2 to < 4	45 (21.3)	52 (24.8)
≥ 4	166 (78.7)	158 (75.2)
HER2 staining fraction, *n* (%)	221 (100.0)	223 (100.0)
Focal (< 30%)	15 (6.8)^b^	16 (7.2)
Heterogeneous (30–79%)	27 (12.2)	27 (12.1)
Homogeneous (≥ 80%)	179 (81.0)	180 (80.7)
HER2 mRNA expression^c^	213 (96.4)	217 (97.3)
Median (range)	36.3 (1.7–1606.8)	35.3 (2.0–548.8)
≤ median, *n* (%)	106 (49.8)	109 (50.2)
> median, *n* (%)	107 (50.2)	108 (49.8)
HER3 mRNA expression^c^	212 (95.9)	218 (97.8)
Median (range)	0.3 (0–2.0)	0.3 (0–2.4)
≤ median, *n* (%)	101 (47.6)	116 (53.2)
> median, *n* (%)	111 (52.4)	102 (46.8)
*PIK3CA* mutation status, *n* (%)	213 (96.4)	212 (95.1)
Mutated	53 (24.9)	61 (28.8)
Non-mutated	160 (75.1)	151 (71.2)
PAM50 subtype, *n* (%)	171 (77.4)	183 (82.1)
HER2-E	104 (60.8)	90 (49.2)
Luminal A	25 (14.6)	35 (19.1)
Luminal B	32 (18.7)	42 (23.0)
Basal-like	10 (5.8)	16 (8.7)
PD-L1, *n* (%)	200 (90.5)	196 (87.9)
IC 0	117 (58.5)	106 (54.1)
IC 1, 2, 3	83 (41.5)	90 (45.9)

*BC* breast cancer, *HER2* human epidermal growth factor receptor 2, *HER2-E* HER2-enriched, *IHC* immunohistochemistry, *ISH* in situ hybridization, *mRNA* messenger RNA, *PAM50* Prediction Analysis of Microarray with the 50-gene classifier, *PIK3CA* phosphatidylinositol-4,5-bisphosphate 3-kinase catalytic subunit alpha, *PD-L1* programmed death ligand 1, *qRT-PCR* quantitative reverse transcriptase polymerase chain reaction, *TCH + P* docetaxel, carboplatin, and trastuzumab plus pertuzumab, *T-DM1 + P* trastuzumab emtansine plus pertuzumab

^a^Two IHC2+ cases were protocol violations as HER2-positive status could not be confirmed centrally

^b^Included two patients with HER2-negative BC

^c^Derived from qRT-PCR assays

### pCR rates by HER2 biomarker subgroups and PIK3CA status

**Fig. 1:**
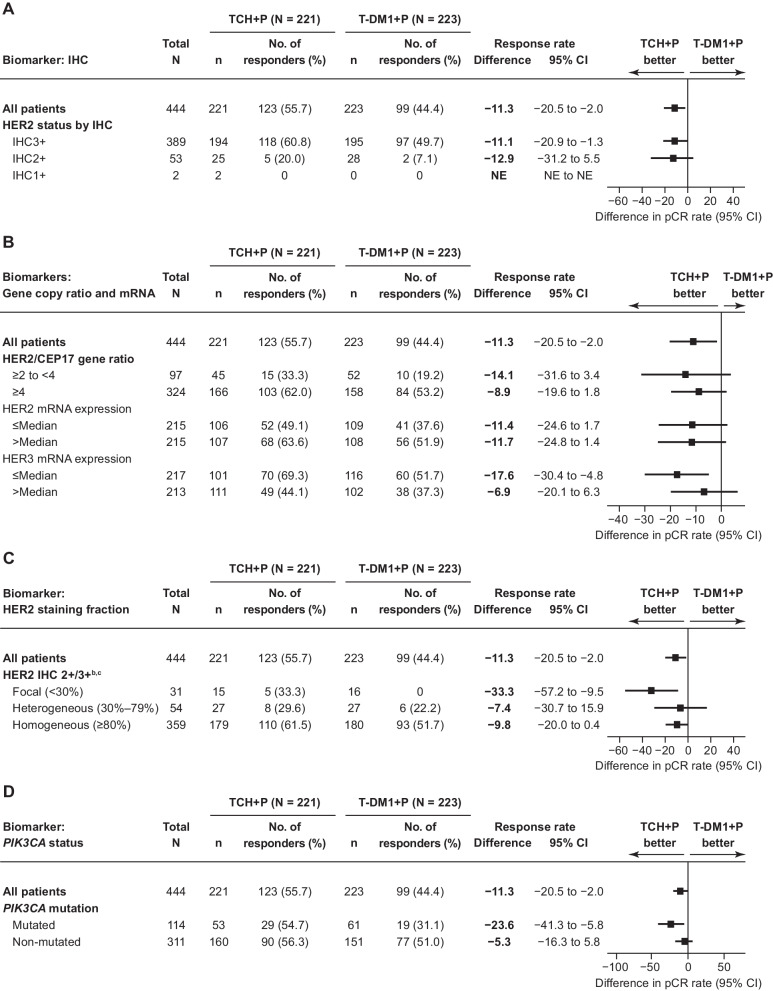
pCR rates^a^ in HER2 biomarker subgroups. **A** HER2 IHC staining percentage; **B** HER2/CEP17 gene ratio and HER2 mRNA level; **C** the pattern of HER2 staining^b^; and **D** *PIK3CA* status. ^a^All unevaluable patients or patients with missing response data were considered to be nonresponders (TCH + P, *n* = 7 [3.2%]; T-DM1 + P, *n* = 18 [8.1%]). ^b^Categorization based on sum of IHC2+ and IHC3+ staining percentages. ^c^Includes two patients with HER2-negative BC. *BC* breast cancer, *CI* confidence interval, *HER2* human epidermal growth factor receptor 2, *IHC* immunohistochemistry, *mRNA* messenger RNA, *NE* not evaluable, *pCR* pathologic complete response, *PIK3CA* phosphatidylinositol-4,5-bisphosphate 3-kinase catalytic subunit alpha, *TCH + P* docetaxel, carboplatin, and trastuzumab plus pertuzumab, *T-DM1 + P* trastuzumab emtansine plus pertuzumab

As previously described, pCR was achieved in 123/221 (55.7%) patients in the TCH-P arm and 99/223 (44.4%) patients in the T-DM1 + P arm (absolute difference −11.3 percentage points, 95% CI −20.5 to −2.0; *P* = 0.016) [10]. Consistent with these data, numerically higher pCR rates were observed for TCH + P versus T-DM1 + P in the majority of the biomarker subgroups in this analysis, including all HER2 pathway biomarker subgroups (Fig. 1). All biomarker subgroups associated with higher HER2 amplification or expression (HER2 IHC3+, HER2/CEP17 gene ratio ≥ 4, HER2 mRNA expression > median, and homogeneous HER2 IHC expression) showed numerically higher pCR rates compared with subgroups with lower HER2 levels, irrespective of treatment arm (Fig. 1A–C). In each of these subgroups, the absolute difference in pCR rates between treatment arms was generally consistent, ranging from − 8.9 to − 11.7 percentage points. Furthermore, high (> median) versus low (≤ median) HER3 mRNA expression showed numerically lower pCR rates in both treatment arms.

In addition, mutated (vs. non-mutated) *PIK3CA* tumors achieved numerically lower pCR rates in the T-DM1 + P arm (although this difference was smaller in the TCH + P arm; Fig. 1D). In the *PIK3CA*-mutated subgroup, a higher proportion of patients treated with T-DM1 + P exhibited low HER2 staining (IHC2+) compared with the TCH + P arm (16.4% [10/61] vs. 7.5% [4/53], respectively, although the sample sizes were small [data not shown]).

### pCR rates by intrinsic tumor subtypes

PAM50 results were available for 77.4% (171/221) of patients in the TCH + P arm and 82.1% (183/223) in the T-DM1 + P arm (Table 1). The most commonly occurring baseline intrinsic subtype was HER2-E (54.8% [194/354]). Luminal A, luminal B, and basal-like subtypes occurred in 16.9% (60/354), 20.9% (74/354), and 7.3% (26/354) of patients, respectively (Additional file 1: Fig. S3A). In patients with known PAM50 subtype, 56.5% (200/354) and 40.4% (143/354) had centrally tested hormone receptor-positive and hormone receptor-negative tumors, respectively; hormone receptor status was unknown for 3.1% (11/354) of patients. The HER2-E subtype was more prevalent in the hormone receptor-negative group versus the hormone receptor-positive group (86.0% [123/143] vs. 32.0% [64/200]). Luminal tumors were more common in patients with hormone receptor-positive (65.5%) versus hormone receptor-negative (0.7%) disease. Most basal-like tumors were identified in the hormone receptor-negative group (Additional file 1: Fig. S3B).

**Fig. 2:**
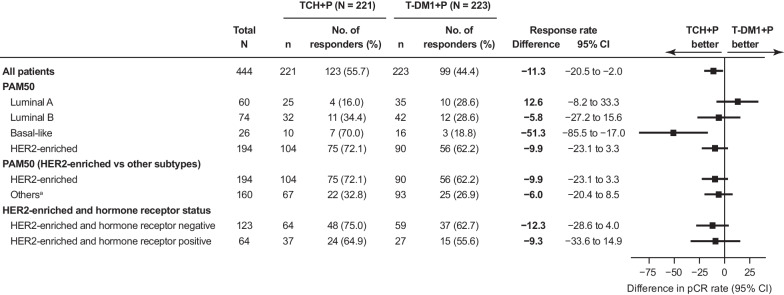
pCR by PAM50 breast cancer subtype and hormone receptor status. ^a^Other subtypes combined included the luminal A, luminal B, and basal-like subtypes. *CI* confidence interval, *HER2* human epidermal growth factor receptor 2, *PAM50* Prediction Analysis of Microarray with the 50-gene classifier, *pCR* pathologic complete response, *TCH + P* docetaxel, carboplatin, and trastuzumab plus pertuzumab, *T-DM1 + P* trastuzumab emtansine plus pertuzumab

Consistent with the results from the intent-to-treat (ITT) population [10], pCR rates were numerically higher in the TCH + P arm versus T-DM1 + P in all subtypes except for luminal A (Fig. 2). In both treatment arms, patients with the HER2-E subtype had the highest pCR rates compared with the other three subtypes, individually or combined. The higher pCR rates in the HER2-E subtype were observed irrespective of hormone receptor status (Fig. 2).

### pCR rates by immune biomarkers

**Fig. 3:**
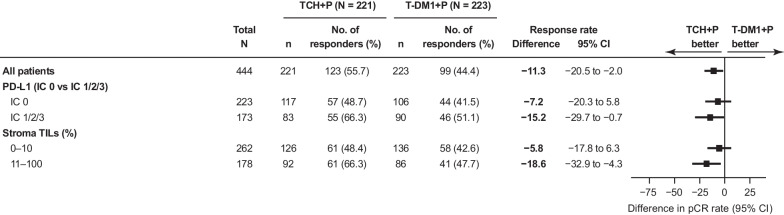
pCR rates by PD-L1 status and stromal TIL level. *CI* confidence interval, *IC* immune cell, *pCR* pathologic complete response, *PD-L1* programmed death ligand 1, *TCH + P* docetaxel, carboplatin, and trastuzumab plus pertuzumab, *T-DM1 + P* trastuzumab emtansine plus pertuzumab, *TIL* tumor-infiltrating lymphocyte

PD-L1 positivity, as assessed on immune cells (IC 1, 2, or 3), was detected in 43.7% (173/396) of patient samples. Most tumors were IC 1 (26%), followed by IC 2 (14%) and IC 3 (4%). pCR rates were numerically higher in the TCH + P versus T-DM1 + P arm, regardless of PD-L1 status (Fig. 3). Additionally, pCR rates were numerically higher in the PD-L1-positive versus PD-L1-negative subgroup in both treatment arms (Fig. 3).

Hormone receptor-negative tumors had a higher prevalence of PD-L1-positive status than hormone receptor-positive tumors (51.7% [78/151] vs. 38.3% [90/235]; Additional file 1: Fig. S4). In patients with hormone receptor-positive disease, pCR rates were numerically higher in the PD-L1-positive subgroup only in the TCH + P treatment arm. In the T-DM1 treatment arm, pCR rates were numerically lower in the PD-L1-positive subgroup of hormone receptor-positive disease. In patients with hormone receptor-negative disease treated with TCH + P, pCR rates were 73.7% for both PD-L1-positive and PD-L1-negative disease. However, pCR rates in the T-DM1 + P arm were numerically higher for PD-L1-positive than PD-L1-negative disease. A forest plot for PD-L1 status per hormone receptor subgroup was generated (Additional file 1: Fig. S4).

Stromal TILs ≤ 10% and > 10% were detected in 59.5% (262/440) and 40.5% (178/440) of patient samples, respectively. pCR rates were numerically higher in the TCH + P versus T-DM1 + P arm across stromal TIL subgroups and higher in patients with stromal TILs > 10% versus ≤ 10% in both treatment arms (Fig. 3).

Results from the RNA sequencing analysis of immune markers were similar to those observed in the ITT population for all immune marker subgroups (Additional file 1: Fig. S5). The treatment benefit was also consistent with that observed in the ITT population.

An analysis of EFS showed long-term trends generally consistent with those obtained from the analysis of pCR, including numerically better EFS in patients with HER2 IHC3+ (vs. HER2 IHC2+), PD-L1-positive (vs. PD-L1-negative) disease, and HER2-E subtype (vs. non-HER2-E) (Additional file 1: Appendix and Table S3).

### Univariate analysis of biomarker association with pCR

**Fig. 4:**
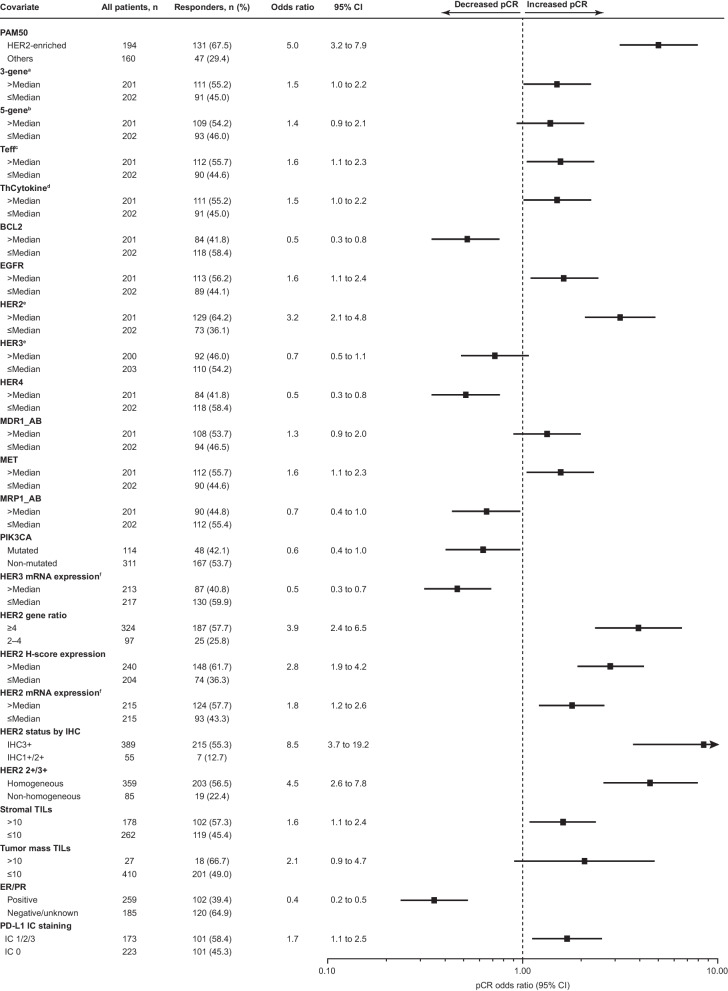
Biomarkers with *P* < 0.15 from a univariate analysis for association with pCR. 3-gene: PD-L1/IFNG/CXCL9 > median versus ≤ median. 5-gene: PD-L1/granzymeB/CD8/IFNG/CXCL9 > median versus ≤ median. Teff: CD8/granzymeA/granzymeB/perforin/IFNG > median versus ≤ median. ThCytokine: CXCL9/CXCL10/CXCL11 > median versus ≤ median. Derived from Nanostring. Derived from qRT-PCR assays. *BCL-2* B cell lymphoma 2, *CD* cluster of differentiation, *CI* confidence interval, *CXCL* C-X-C motif chemokine ligand, *EGFR* epidermal growth factor receptor, *ER* estrogen receptor, *HER* human epidermal growth factor receptor, *IC* immune cell, *IFNG* interferon gamma, *IHC* immunohistochemistry, *MDR1* multidrug resistance mutation 1, *MET* MET protooncogene, receptor tyrosine kinase, *mRNA* messenger RNA, *MRP1* multidrug resistance–associated protein 1, *PAM50* Prediction Analysis of Microarray with the 50-gene classifier, *pCR* pathologic complete response, *PD-L1* programmed death ligand 1, *PIK3CA* phosphatidylinositol-4,5-bisphosphate 3-kinase catalytic subunit alpha, *PR* progesterone receptor, *qRT-PCR* quantitative reverse transcriptase polymerase chain reaction, *Teff* T effector, *ThCytokine* chemokine signaling, *TIL* tumor-infiltrating lymphocyte

A univariate analysis of all biomarkers, including genes and signatures of interest from the NanoString panel, was conducted to evaluate their prognostic value for pCR (Additional file 1: Table S2). Of these biomarkers, 24 were selected for further multivariate analyses based on a *P* value < 0.15 (Fig. 4). Selected biomarkers with a positive association with pCR included homogeneous HER2 IHC3+ intensity; HER2 mRNA expression > median by both qRT-PCR and Nanostring; HER2 H-score ≥ median; HER2 gene ratio ≥ 4; PAM50 HER2-E subtype; PD-L1-positive IHC status; stromal TILs > 10%; tumor mass TILs > 10%; and immune gene signatures, epidermal growth factor receptor (EGFR), MDR1/ABCB1, and MET gene expression > median. Biomarkers with a negative association with pCR included *PIK3CA* mutation; hormone receptor-positive status; HER3 mRNA > median by both qRT-PCR and Nanostring; HER4; BCL-2; and multidrug resistance-associated protein 1 (MRP1) > median. Fcy polymorphisms did not impact pCR.

### Multivariate analysis of biomarker association with pCR

After applying backwards selection, three biomarker covariates from the univariate analysis (PAM50 HER2-E subtype, HER2 gene ratio ≥ 4, and PD-L1-positive IHC status) were positively associated with pCR (Table 2) and were included in a final logistic model, along with the randomized treatment arms. A total of 314 patients (71% of the ITT population) with no missing covariates were included in the final model.

**Table 2:** Multivariate analysis of treatment effect and selected biomarkers associated with pCR

Effect	Degrees of freedom^a^	Odds ratio (95% CI)
Treatment effect TCH + P (reference; *n* = 160) versus T-DM1 + P (*n* = 154)	1	0.68 (0.41–1.12)
PAM50 intrinsic subtypes Other subtypes (reference; *n* = 137) versus HER2-E (*n* = 177)	1	4.02 (2.41–6.70)
HER2 gene ratio 2–4 (reference; *n* = 71) versus ≥ 4 (*n* = 243)	1	4.08 (2.12–7.84)
PD-L1 IHC staining subgroups IC 0 (reference; *n* = 172) versus IC 1, 2, 3 (*n* = 142)	1	2.48 (1.48–4.14)

Analysis conducted in patients with no missing covariates

The final logistic model included 314 patients

*CI* confidence interval, *HER2* human epidermal growth factor receptor 2, *HER2-E* human epidermal growth factor receptor 2-enriched, *IC* immune cell, *IHC* immunohistochemistry, *PAM50* Prediction Analysis of Microarray with the 50-gene classifier, *pCR* pathologic complete response, *PD-L1* programmed death ligand 1, *TCH + P* docetaxel, carboplatin, and trastuzumab plus pertuzumab, *T-DM1 + P* trastuzumab emtansine plus pertuzumab

^a^Degrees of freedom represent the number of categories used versus the reference

### Change from baseline in biomarkers of interest

Changes in biomarkers at baseline, cycle 2, and surgery were assessed. These findings are exploratory, as the number of samples available post-baseline was limited and patients who did not achieve pCR were more likely to have biomarker results at cycle 2 (71.5% of tissue availability for non-pCR subgroup vs. 28.5% for pCR subgroup). PAM50 intrinsic tumor subtype, PD-L1-positive IHC status, percentage of stromal TILs, and HER2 gene expression levels (Nanostring) were analyzed by the treatment arm at different time points. HER2-E tumors were the most prevalent PAM50 subtype in both treatment arms at baseline (Additional file 1: Fig. S6A). In the TCH + P arm, the proportion of HER2-E tumors significantly decreased over time, which resulted in luminal A tumors being the most common at cycle 2 and surgery. In the T-DM1 + P arm, the proportion of HER2-E tumors also decreased, although to a lesser extent than in the TCH + P arm. At cycle 2 and surgery, the luminal A and B tumor types were the most common, accounting for slightly more than 50% of patients. In both treatment arms, the proportion of patients with HER2 gene expression > median decreased from baseline to surgery (Additional file 1: Fig. S6B).

PD-L1-positive IHC status was observed in similar percentages of patients at baseline (41.5%) and cycle 2 (37.3%) in the TCH + P arm, decreasing to 21.4% at surgery. In the T-DM1 + P arm, in contrast, PD-L1 positivity increased from 45.9% at baseline to 53.8% at cycle 2 and 72.5% at surgery (Additional file 1: Fig. S6C). However, dynamic changes in on-treatment PD-L1 status were observed for both the TCH + P (Additional file 1: Fig. S6D) and T-DM1 + P (Additional file 1: Fig. S6E) arms from baseline to cycle 2. The proportion of patients with stromal TILs > 10% increased from baseline to cycle 2, but decreased between cycle 2 and surgery in both treatment arms (Additional file 1: Fig. S6F). This decrease was more evident in the TCH + P arm (42.2% at baseline, increasing to 56.5% at cycle 2 and decreasing to 23.1% at surgery). For immune markers and signatures, there was no trend toward difference between treatment arms, and the change from baseline was modest with a trend toward increased expression at neoadjuvant cycle 2 for most immune signatures (Additional file 1: Fig. S7).

### Co-occurrence of biomarkers

HER2-related biomarkers (HER2-E subtype, high gene ratio, homogeneous staining, high H-score, and IHC3+ status) were all highly correlated with each other, as shown in the balloon plot in Additional file 1: Fig. S8. Immune-related biomarkers (PD-L1-positive status, high PD-L1/CD8/Teff gene/Teff signature expression levels, and high TILs) were also shown to co-occur. No clear and consistent association between high levels of HER2- and immune-related markers was observed, apart from some weak single associations (e.g., HER2-E with high stromal TILs; in contrast with high HER2 mRNA with low PD-L1, low Teff mRNA, and low stromal TILs). High HER3 mRNA showed a negative association with high levels of both HER2- and immune-related markers, except a positive association between high HER3 mRNA and high HER2 mRNA.

Other markers showing a negative association with high HER2-related biomarkers were hormone receptor-positive status (all with the exception of HER2 H-score), *PIK3CA* mutation (with high HER2 H-score and high HER2 mRNA), and high bcl-2 expression (with HER2-E subtype, homogeneous HER2, and HER2 IHC3+).

Interestingly, a positive hormone receptor status was more likely to be associated with low immune marker expression and to co-occur with high bcl-2 and high HER3 RNA expression. Mutated *PIK3CA* was shown to correlate with low HER3 mRNA levels and high PD-L1 gene expression, but with no other immune markers.

## Discussion

Consistent with the analysis of pCR in the ITT population from KRISTINE [10], pCR rates in this exploratory biomarker analysis were numerically lower in the T-DM1 + P versus TCH + P treatment arm for the majority of the assessed biomarkers. The only exception was the luminal A subgroup, which appeared to favor T-DM1 + P; however, the interpretation of these data is limited by the small sample sizes in this subgroup. Therefore, no subgroup of patients that clearly benefited from neoadjuvant T-DM1 + P versus TCH + P was identified. As expected, higher HER2 levels (based on mRNA and protein expression, gene amplification, and HER2-E intrinsic subtype) were associated with numerically higher pCR rates versus those observed with lower HER2 levels, irrespective of treatment arm. Additionally, immune activation factors, including PD-L1 positivity, higher immune gene expression, and higher TILs, were associated with higher pCR rates; this was especially notable in the TCH + P arm. These data are consistent with previous reports from neoadjuvant breast cancer studies of HER2-targeted therapy [16, 24, 25, 27, 37, 38] and recently reported validation results from the HER2DX assay, which integrates HER2 subtype information, immune information, and ERBB2 levels into a single score [39].

The multivariate analysis indicated that PAM50 HER2-E subtype, HER2 gene ratio ≥ 4, and PD-L1-positive IHC status positively influenced the pCR rate, while hormone receptor-negative status was not shown to be a covariate influencing the pCR rate. However, patients with hormone receptor-negative disease were more likely to be PD-L1-positive than patients with hormone receptor-positive disease. Similar to previous studies [8, 40, 41], hormone receptor-negative status was associated with a higher pCR rate, but did not remain a significant factor after correcting for other factors. The reason for the difference in pCR rate between treatment arms in the PD-L1-negative versus PD-L1-positive subgroups within the HR subgroups is unclear. It should be noted that as these are small subsets within a subgroup analysis, no firm conclusions can be drawn.

Previous studies assessing the impact of *PIK3CA* mutation status on HER2-targeted treatments for eBC appear to have been influenced by the study setting and/or treatments received. In the neoadjuvant eBC setting, *PIK3CA* mutations have generally been associated with worse outcomes, including lower pCR rates, in patients treated with HER2-targeted therapies [17, 42]. However, the impact of *PIK3CA* mutations on long-term outcomes in the adjuvant setting is less clear [43–45]. In our analysis of data from KRISTINE, *PIK3CA* mutations were associated with a lower pCR rate in a univariate analysis, although this effect was mainly present in the T-DM1 + P arm. These results differ from previous studies in second-line mBC that did not show an association between *PIK3CA* mutation and efficacy in T-DM1-treated patients with HER2-positive breast cancer, suggesting that T-DM1 treatment may overcome the prognostic value of *PIK3CA* mutations [13]. The differences between the current study and previous analyses may also be due to imbalances in other influencing factors—such as HER2 IHC or hormone receptor status—within these subgroups across treatment arms, as well as to differences in treatment lines and disease setting. Notably, the multivariate analysis did not confer a clear prognostic value for pCR rates for *PIK3CA* mutations.

In addition, we observed that hormone receptor-positive disease was negatively associated with almost all HER2-related biomarkers with high levels of HER2 expression, and *PIK3CA* mutation showed a negative association with high HER2 H-score and mRNA. This may have contributed to the fact that *PIK3CA* and hormone receptor status were not included in the final multivariate model. Other explanations include the relatively small sample size and differences in the impact of the biomarker on pCR between the two treatment arms. The balloon plot further shows the independent presence of high HER2 and high immune markers in the tumors and may therefore explain the role of the HER2-E subtype, HER2 gene ratio, and PD-L1 as independent factors associated with pCR.

We observed alterations in “on-treatment” biomarkers from baseline to the time of surgery. The proportion of luminal A tumor subtypes, previously identified as predictive of resistance to anti-HER2 therapy [46], increased in both “on-treatment” and “post-treatment” tumor samples. The increased proportion of the luminal A subtype and subsequent decreased proportion of the HER2-E subtype over time was predominantly observed in the TCH + P group; this suggests that there is a shift in tumor cells with characteristics that may be less sensitive to combination treatment with systemic chemotherapy and HER2-targeted therapy or that the increase in the prevalence of luminal A subtype is simply due to a more prominent reduction in the number of HER2-positive tumor cells within more heterogeneous tumors. The rapid shift to a higher proportion of luminal disease subtypes with dual HER2 blockade observed in this study was also reported in the CALGB and PAMELA trials, highlighting the fluidity of molecular subtyping under treatment pressure [46, 47]. While a general conversion of subtypes was seen in 36% of patients who progressed from primary to metastatic disease in the AURORA study [48], the change from HER2-E into luminal subtypes was rarely observed, suggesting that the HER2-E subtype may be more stable. Of note, the AURORA data were generated across breast cancer types, not only HER2-positive disease. Another explanation for the differences in subtype fluidity over time and disease settings may be the role of adjuvant endocrine treatment, which is absent in the neoadjuvant setting. An additional finding in KRISTINE was that in the TCH + P arm, the proportion of PD-L1-positive tumors was similar from baseline to cycle 2, but decreased from baseline to surgery. In contrast, PD-L1-positivity increased between baseline and cycle 2, as well as between cycle 2 and surgery, in the T-DM1 + P arm. Thus, while an overall increase in PD-L1-positivity was mainly seen in the T-DM1 + P arm, dynamic changes in PD-L1 status during treatment were observed in both treatment arms. The changes in PD-L1 expression likely represent increased immune activation resulting from therapy, but more data are needed on potential differences in immune stimulation relating to the type of chemotherapy used (DM1 versus docetaxel/carboplatin), the route of administration (systemic versus targeted delivery via an ADC), and the role of preexisting immunity [49]. Overall, these data suggest that immune stimulation can be induced by both traditional chemotherapy and an ADC (i.e., T-DM1), perhaps as a result of antigen spreading induced by antigens released from dying tumor cells.

These changes in “on-treatment” biomarkers align with findings in the neoadjuvant setting, including studies of chemotherapy combined with HER2-targeted therapies [46, 47]. In the TRIO-US B07 phase II trial, immune signature levels decreased at the time of surgery in patients with HER2-positive breast cancer treated with neoadjuvant trastuzumab and/or lapatinib plus chemotherapy [37]. In a study of anti-HER2-based neoadjuvant treatment in HER2-positive breast cancer, stromal TILs consistently decreased at surgery in patients who achieved pCR, whereas most residual tumors remained inflamed at surgery [50].

Our biomarker analysis has several limitations. The sample sizes for some subgroups were small, particularly for the breast cancer subtype, biomarker co-occurrence, subsets of subgroups, and post-baseline time points. The small number of EFS events and the receipt of other therapies (e.g., chemotherapy, hormonal therapy) following neoadjuvant treatment in some patients limit the potential impact of a biomarker analysis for this clinical outcome. A numerically higher rate of the HER2-E subtype in the TCH + P arm may have impacted overall results due to the association between HER2 subtype and increased pCR rate. Only three biomarkers of interest (HER2, PD-L1 IHC status, and *PIK3CA* mutation) were pre-specified to be included in this analysis and were investigated following the pooling of data from both treatment arms. Therefore, no firm conclusions should be drawn. Of note, 15 EFS events were reported during the neoadjuvant period in the T-DM1 + P arm, and these were associated with low and heterogeneous HER2 expression [11]. Additionally, interpretation is limited by the enrichment of patients without pCR in biomarker data derived from serial samples. Again, firm conclusions cannot be made regarding data reported for changes from baseline in PAM50 intrinsic subtypes, PD-L1 expression, TILs, and HER2 mRNA, as these may be biased by the fact that patients with residual disease were more likely to have tumor tissue available at cycle 2 as well as at surgery.

## Conclusion

Our analysis of biomarkers in the KRISTINE study confirms the role of HER2 and the immune microenvironment in achieving pCR after dual HER2-targeted therapy, with the PAM50 HER2-E subtype, HER2 gene ratio ≥ 4, and PD-L1-positive IHC status being positively associated with pCR. HER2 IHC3+ and PD-L1-positive status were associated with numerically better EFS compared with HER2 IHC2+ and PD-L1-negative status. The observed associations between PD-L1 and clinical outcomes may be related more to their independent prognostic potential than to a relationship with response to HER2-targeted therapeutics, as similar associations have been observed in studies of patients with triple-negative breast cancer [51, 52]. Additionally, high levels of HER2 and immune-related markers may impact tumors independently, as suggested by the multivariate analysis and the lack of co-occurrence. Dynamic changes were observed on-treatment for paired biopsy analyses of select biomarkers. The overlap of biomarkers unfavorable to pCR, such as positive hormone receptor status, *PIK3CA* mutation, low HER2 levels, and non HER2-E subtypes, reaffirms the complex molecular diversity of HER2-positive breast tumors and should be considered when designing clinical trials of HER2-targeted treatments for early-stage HER2-positive breast cancer.

## Supplementary Information

**Additional file 1**: de Haas_KRISTINE biomarkers manuscript_Appendix.docx (supplementary results, discussion, tables, and figures).

## Data Availability

For up-to-date details on Roche's Global Policy on the Sharing of Clinical Information and how to request access to related clinical study documents, see https://go.roche.com/data_sharing. Qualified researchers may request access to de-identified patient-level data and clinical study documentation via the following link: http://www.roche.com/research_and_development/who_we_are_how_we_work/clinical_trials/our_commitment_to_data_sharing/clinical_study_documents_request_form.htm. Anonymized records for individual patients across more than one data source external to Roche cannot, and should not, be linked due to a potential increase in the risk of patient re-identification.

## Abbreviations

ADC: Antibody–drug conjugate
CI: Confidence interval
eBC: Early breast cancer
EFS: Event-free survival
FDA: US Food and Drug Administration
HER2: Anti-human epidermal growth factor receptor 2
HER2-E: HER2-enriched
HR: Hazard ratio
IC: Immune cell
iDFS: Invasive disease-free survival
IHC3+: Immunohistochemistry 3+
ITT: Intent-to-treat
mBC: Metastatic breast cancer
*MDR1*: Multidrug resistance 1
mRNA: Messenger RNA
PAM50: Prediction Analysis of Microarray with the 50-gene classifier
PD-L1: Programmed death ligand 1
*PIK3CA*: Phosphatidylinositol-4,5-bisphosphate 3-kinase catalytic subunit alpha
qRT-PCR: Quantitative reverse transcriptase polymerase chain reaction
TCH + P: Docetaxel, carboplatin, and trastuzumab plus pertuzumab
T-DM1: Trastuzumab emtansine
T-DM1 + P: T-DM1 alone or in combination with pertuzumab
Teff: T effector
TIL: Tumor-infiltrating lymphocyte
