# Supplementary material for: Tumor biomarkers and efficacy in patients treated with trastuzumab emtansine + pertuzumab versus standard of care in HER2-positive early breast cancer: an open-label, phase III study (KRISTINE)
Source: Breast Cancer Res. 2023 Jan 11;25:2. doi: 10.1186/s13058-022-01587-z (PMC9832665; doi:10.1186/s13058-022-01587-z)
Supplement: Supplementary file 1 — Additional file 1: de Haas_KRISTINE biomarkers manuscript_Appendix.docx (supplementary results, discussion, tables, and figures). [file 13058_2022_1587_MOESM1_ESM.docx]

**Appendix**

**Supplementary Results**

*Association of biomarkers with event-free survival (EFS)*

The potential long-term prognostic value of HER2 immunohistochemistry (IHC) subgroups, PD-L1 status by IHC, and *PIK3CA* mutation status on EFS was assessed using data pooled from both treatment arms. With the caveat that the number of EFS events was limited, HER2 IHC3+, PD-L1–positive status, and HER2-E subtype were all associated with a trend toward better EFS compared with HER2 IHC2+ (unstratified hazard ratio [HR] 0.23, 95% CI 0.12–0.44), PD-L1–negative status (unstratified HR 0.55, 95% CI 0.27–1.11), and non-HER2-E subtype (unstratified HR 0.39, 95% CI 0.20–0.76), respectively (Table S3). No prognostic value was observed for mutated versus non-mutated *PIK3CA* status (unstratified HR 1.34, 95% CI 0.71–2.53). Overall, these trends are consistent with those obtained from the analysis of pCR.

**Supplementary Discussion**

The analysis of long-term outcomes suggested that patients with PD-L1–positive tumors, HER2 IHC3+ status, and HER2-E subtype prior to neoadjuvant treatment trended towards improved EFS. Higher HER2 expression in pre-treatment samples has previously been associated with better EFS in patients receiving neoadjuvant HER2-targeted therapy (1), but individual neoadjuvant trials are limited due to small sample sizes and have limited power to show correlation with long-term outcomes. However, the association of higher HER2 levels with better clinical outcomes should be further explored, given current interest in the de-escalation of chemotherapy for patients with HER2-positive eBC. Although EFS was lower in the T-DM1+P arm, there may be benefit to using a chemotherapy-sparse regimen with T-DM1 and/or other HER2-targeted therapies to spare patients from the adverse effects of chemotherapeutic agents, particularly those with high HER2 amplification or expression at baseline (2). Pooling data from multiple trials could provide further insights, as shown in a study by Prat et al (3) where combining HER2-E subtype and high HER2 mRNA led to the identification of tumors with high responsiveness to HER2-targeted therapy. Future studies can further elucidate the potential role of neoadjuvant chemotherapy-sparse regimens with T-DM1 and/or other HER2-targeted therapies for HER2-positive disease.

**Supplementary References**

1. Prat A, Bianchini G, Thomas M, Belousov A, Cheang MCU, Koehler A, et al. Research-based PAM50 subtype predictor identifies higher responses and improved survival outcomes in HER2-positive breast cancer in the NOAH study. Clin Cancer Res. 2014;20:511–21.
2. Hurvitz SA, Martin M, Jung KH, Huang CS, Harbeck N, Valero V, et al. Neoadjuvant trastuzumab emtansine and pertuzumab in human epidermal growth factor receptor 2-positive breast cancer: three-year outcomes from the phase III KRISTINE study. J Clin Oncol. 2019;37(25):2206–16.
3. Prat A, Pascual T, De Angelis C, Gurierrez C, Llombart-Cussac A, Wang T, et al. HER2-enriched subtype and ERBB2 expression in HER2-positive breast cancer treated with dual HER2 blockage. J Natl Cancer Inst. 2020;112:46–54.

**Table S1. Biomarkers evaluated in the KRISTINE study.**

| **Biomarkers** | |
| --- | --- |
| HER2 ISH/IHC status | |
| Central hormone receptor status by IHC | |
| *PIK3CA* mutation status | |
| Other HER family biomarkers  HER2 mRNA expression^a^  HER3 mRNA expression^a^  HER2 gene ratio  HER2 H-score  HER2 staining fraction | |
| Nanostring breast cancer panel: | |
| PAM50 intrinsic subtypes  Immune genes  PD-L1 expression  CD8 expression  Immune signatures  3-gene^b^  5-gene^c^  Teff^d^  ThCytokines^e^  Checkpoint inhibitors^f^  FcyR and ABCB1 polymorphisms  FCγRIIA  FCγRIIIA  ABCB1-polymorphism  ABCG2-polymorphism | Other candidates  APOBEC1  APOBEC3B  BCL2  BCLXL  BCRP_AB  C8A  EGFR  HER2  HER3  HER4  MCL1  MDR1 (ABCB1)  MET  MRP1 (ABCC1)  MRP4  NMU  NRG1  POSTN  PTEN |
| PD-L1 status by IHC (immune cells)^g^ | |
| Tumor-infiltrating lymphocytes ^g^  Stromal  Tumor mass | |

^a^Derived from qRT-PCR assays.

^b^3-gene: PD-L1/IFNG/CXCL9 >median vs. ≤median.

^c^5-gene: PD-L1/granzymeB/CD8/IFNG/CXCL9 >median vs. ≤median.

^d^Teff: CD8/granzymeA/granzymeB/perforin/IFNG >median vs. ≤median.

^e^ThCytokine: CXCL9/CXCL10/CXCL11 >median vs. ≤median.

^f^Checkpoint inhibitors: PD-L1/PD-L2/IDO >median vs. ≤median.

^g^Included post hoc.

Abbreviations: BCL2, B-cell lymphoma 2; BCRP, breast cancer resistance protein; CD, cluster of differentiation; CXCL, C-X-C motif chemokine ligand; EGFR, epidermal growth factor receptor; Fc, fragment crystallizable; H-score, histoscore; HER, human epidermal growth factor receptor; IDO, indoleamine 2,3-dioxygenase; IFNG, interferon gamma; IHC, immunohistochemistry; ISH, in situ hybridization; MCL1, myeloid cell leukemia-1; MDR1, multidrug resistance 1; MET, MET protooncogene, receptor tyrosine kinase; mRNA, messenger RNA; MRP, multidrug resistance–associated protein; NMU, neuromedin U; NRG1, neuregulin 1; PAM50, Prediction Analysis of Microarray with the 50-gene classifier; PD-L1, programmed death-ligand 1; *PIK3CA*, phosphatidylinositol-4,5-bisphosphate 3-kinase catalytic subunit alpha; POSTN, periostin; PTEN, phosphatase and tensin homolog; qRT-PCR, quantitative reverse transcriptase polymerase chain reaction; TEFF, T effector; ThCytokine, chemokine signaling.

**Table S2. Univariate analysis of pCR by biomarker status in the randomized patient population.**

| **Covariate** | **All patients, n** | **Responders, n (%)** | **Odds ratio (95% CI)** |
| --- | --- | --- | --- |
| *PIK3CA* mutation^a^ |  |  |  |
| Mutated | 114 | 48 (42.1) | 0.6 (0.4–1.0) |
| Non-mutated | 311 | 167 (53.7) |  |
| HER2/HER3 mRNA (by qRT PCR) and gene copy number/ratio |  |  |  |
| HER2 mRNA expression^a^ |  |  |  |
| >Median | 215 | 124 (57.7) | 1.8 (1.2–2.6) |
| ≤Median | 215 | 93 (43.3) |  |
| HER3 mRNA expression^a^ |  |  |  |
| >Median | 213 | 87 (40.8) | 0.5 (0.3–0.7) |
| ≤Median | 217 | 130 (59.9) |  |
| HER2 gene ratio^a^ |  |  |  |
| ≥4 | 324 | 187 (57.7) | 3.9 (2.4–6.5) |
| 2–4 | 97 | 25 (25.8) |  |
| HER2 H-score expression^a^ |  |  |  |
| ≥Median | 240 | 148 (61.7) | 2.8 (1.9–4.2) |
| <Median | 204 | 74 (36.3) |  |
| HER2 IHC status ^a^ |  |  |  |
| IHC3+ | 389 | 215 (55.3) | 8.5 (3.7–19.2) |
| IHC1+/2+ | 55 | 7 (12.7) |  |
| HER2 staining fraction^a^ |  |  |  |
| Homogeneous | 359 | 203 (56.5) | 4.5 (2.6–7.8) |
| Non-homogeneous | 85 | 19 (22.4) |  |
| Central ER/PR status^a^ |  |  |  |
| Positive | 259 | 102 (39.4) | 0.4 (0.2–0.5) |
| Negative/unknown | 185 | 120 (64.9) |  |
| PAM50 intrinsic subtypes^a^ |  |  |  |
| HER2 enriched | 194 | 131 (67.5) | 5.0 (3.2–7.9) |
| Others | 160 | 47 (29.4) |  |
| PD-L1 IHC staining ^a^ |  |  |  |
| IC 1, 2, 3 | 173 | 101 (58.4) | 1.7 (1.1–2.5) |
| IC 0 | 223 | 101 (45.3) |  |
| TILs |  |  |  |
| Stromal TILs^a^ |  |  |  |
| >10 | 178 | 102 (57.3) | 1.6 (1.1–2.4) |
| ≤10 | 262 | 119 (45.4) |  |
| Tumor mass TILs^a^ |  |  |  |
| >10 | 27 | 18 (66.7) | 2.1 (0.9–4.7) |
| ≤10 | 410 | 201 (49.0) |  |
| Immune genes and signatures (by Nanostring) |  |  |  |
| PD-L1 |  |  |  |
| >Median | 201 | 102 (50.7) | 1.1 (0.7–1.6) |
| ≤Median | 202 | 100 (49.5) |  |
| CD8 |  |  |  |
| >Median | 201 | 105 (52.2) | 1.2 (0.8–1.8) |
| ≤Median | 202 | 97 (48.0) |  |
| 3-gene^a^ |  |  |  |
| PD-L1/IFNG/CXCL9 >median | 201 | 111 (55.2) | 1.5 (1.0–2.2) |
| PD-L1/IFNG/CXCL9 ≤median | 202 | 91 (45.0) |  |
| 5-gene^a^ |  |  |  |
| PD-L1/granzymeB/CD8/IFNG/CXCL9 >median | 201 | 109 (54.2) | 1.4 (0.9–2.1) |
| PD-L1/granzymeB/CD8/IFNG/CXCL9 ≤median | 202 | 93 (46.0) |  |
| Teff^a^ |  |  |  |
| CD8/granzymeA/granzymeB/perforin/IFNG >median | 201 | 112 (55.7) | 1.6 (1.1–2.3) |
| CD8/granzymeA/granzymeB/perforin/IFNG ≤median | 202 | 90 (44.6) |  |
| ThCytokine^a^ |  |  |  |
| CXCL9/CXCL10/CXCL11 >median | 201 | 111 (55.2) | 1.5 (1.0–2.2) |
| CXCL9/CXCL10/CXCL11 ≤median | 202 | 91 (45.0) |  |
| Checkpoint inhibitors |  |  |  |
| PD-L1/PD-L2/IDO >median | 201 | 106 (52.7) | 1.2 (0.8–1.8) |
| PD-L1/PD-L2/IDO ≤median | 202 | 96 (47.5) |  |
| Fcγ polymorphisms and ABCB1/G2 polymorphisms |  |  |  |
| FCγR IIA |  |  |  |
| AA | 133 | 64 (48.1) | 0.9 (0.6–1.4) |
| Rest | 285 | 145 (50.9) |  |
| FCγR IIIA |  |  |  |
| CC | 41 | 23 (56.1) | 1.3 (0.7–2.5) |
| Rest | 377 | 186 (49.3) |  |
| ABCB1− RS1045642 |  |  |  |
| GG | 122 | 57 (46.7) | 0.8 (0.6–1.3) |
| Rest | 296 | 152 (51.4) |  |
| ABCG2− RS2231142 |  |  |  |
| GG | 317 | 162 (51.1) | 1.2 (0.8–1.9) |
| Rest | 101 | 47 (46.5) |  |
| Other genes (by Nanostring) |  |  |  |
| APOBEC1 |  |  |  |
| >0 | 86 | 40 (46.5) | 0.8 (0.5–1.3) |
| 0 | 317 | 162 (51.1) |  |
| APOBEC3B |  |  |  |
| >Median | 200 | 104 (52.0) | 1.2 (0.8–1.7) |
| ≤Median | 203 | 98 (48.3) |  |
| BCL2^a^ |  |  |  |
| >Median | 201 | 84 (41.8) | 0.5 (0.3–0.8) |
| ≤Median | 202 | 118 (58.4) |  |
| BCLXL |  |  |  |
| >Median | 201 | 100 (49.8) | 1.0 (0.7–1.4) |
| ≤Median | 202 | 102 (50.5) |  |
| BCRP_AB |  |  |  |
| >Median | 200 | 96 (48.0) | 0.8 (0.6–1.2) |
| ≤Median | 203 | 106 (52.2) |  |
| C8A |  |  |  |
| >Median | 201 | 106 (52.7) | 1.2 (0.8–1.8) |
| ≤Median | 202 | 96 (47.5) |  |
| EGFR^a^ |  |  |  |
| >Median | 201 | 113 (56.2) | 1.6 (1.1–2.4) |
| ≤Median | 202 | 89 (44.1) |  |
| HER2^a^ |  |  |  |
| >Median | 201 | 129 (64.2) | 3.2 (2.1–4.8) |
| ≤Median | 202 | 73 (36.1) |  |
| HER3^a^ |  |  |  |
| >Median | 200 | 92 (46.0) | 0.7 (0.5–1.1) |
| ≤Median | 203 | 110 (54.2) |  |
| HER4^a^ |  |  |  |
| >Median | 201 | 84 (41.8) | 0.5 (0.3–0.8) |
| ≤Median | 202 | 118 (58.4) |  |
| MCL1 |  |  |  |
| >Median | 201 | 99 (49.3) | 0.9 (0.6–1.4) |
| ≤Median | 202 | 103 (51.0) |  |
| MDR1 (ABCB1)^a^ |  |  |  |
| >Median | 201 | 108 (53.7) | 1.3 (0.9–2.0) |
| ≤Median | 202 | 94 (46.5) |  |
| MET^a^ |  |  |  |
| >Median | 201 | 112 (55.7) | 1.6 (1.1–2.3) |
| ≤Median | 202 | 90 (44.6) |  |
| MRP1 (ABCC1)^a^ |  |  |  |
| >Median | 201 | 90 (44.8) | 0.7 (0.4–1.0) |
| ≤Median | 202 | 112 (55.4) |  |
| MRP4 |  |  |  |
| >Median | 201 | 107 (53.2) | 1.3 (0.9–1.9) |
| ≤Median | 202 | 95 (47.0) |  |
| NMU |  |  |  |
| >0 | 232 | 121 (52.2) | 1.2 (0.8–1.8) |
| 0 | 171 | 81 (47.4) |  |
| NRG1 |  |  |  |
| >Median | 201 | 98 (48.8) | 0.9 (0.6–1.3) |
| ≤Median | 202 | 104 (51.5) |  |
| POSTN |  |  |  |
| >Median | 200 | 94 (47.0) | 0.8 (0.5–1.2) |
| ≤Median | 203 | 108 (53.2) |  |
| PTEN |  |  |  |
| >Median | 201 | 98 (48.8) | 0.9 (0.6–1.3) |
| ≤Median | 202 | 104 (51.5) |  |

^a^Indicates variables with *P*<0.15; these covariates were used in step 2 of the multivariate analysis.

Abbreviations: BCL2, B-cell lymphoma 2; CD, cluster of differentiation; CI, confidence interval; CXCL, C-X-C motif chemokine ligand; EGFR, epidermal growth factor receptor; ER, estrogen receptor; Fc, fragment crystallizable; H-score, histoscore; HER, human epidermal growth factor receptor; IC, immune cells; IFNG, interferon gamma; IHC, immunohistochemistry; mRNA, messenger RNA; NMU, neuromedin U; NRG1, neuregulin 1; PAM50, Prediction Analysis of Microarray with the 50-gene classifier; pCR, pathologic complete response; PD-L, programmed death-ligand; *PIK3CA*, phosphatidylinositol-4,5-bisphosphate 3-kinase catalytic subunit alpha; POSTN, periostin; PR, progesterone receptor; qRT-PCR, quantitative reverse transcriptase polymerase chain reaction; TEFF, T effector; ThCytokine, chemokine signaling; TILs, tumor-infiltrating lymphocytes.

**Table S3. Prognostic analysis: Event-free survival by HER2 IHC, PD-L1 IHC, and *PIK3CA* mutation status (pooled treatment arms).**

|  | **HER2 IHC** | | **PD-L1 IHC** | | ***PIK3CA*** | | **PAM50 subtype** | |
| --- | --- | --- | --- | --- | --- | --- | --- | --- |
|  | **2+**  **(*n*=53)** | **3+**  **(*n*=389)** | **IC 0**  **(*n*=223)** | **IC 1/2/3**  **(*n*=173)** | **Non-mutated**  **(*n*=312)** | **Mutated**  **(*n*=114)** | **Non-HER2-E**  **(*n*=160)** | **HER2-E**  **(*n*=194)** |
| Patients with event, *n* (%) | 14 (26.4) | 30 (7.7) | 25 (11.2) | 11 (6.4) | 29 (9.3) | 14 (12.3) | 25^a^ (15.6) | 13 (6.7) |
| Earliest contributing event, *n* |  |  |  |  |  |  |  |  |
| Disease progression/relapse | 14 | 26 | 22 | 10 | 25 | 14 | 22 | 12 |
| Deaths without prior EFS event | 0 | 4 | 3 | 1 | 4 | 0 | 3 | 1 |
| Patients without event, *n* (%) | 39 (73.6) | 359 (92.3) | 198 (88.8) | 162 (93.6) | 283 (90.7) | 100 (87.7) | 135 (84.4) | 181 (93.3) |
| Unstratified HR (95% CI) | 0.23 (0.12–0.44) | | 0.55 (0.27–1.11) | | 1.34 (0.71–2.53) | | 0.39 (0.20–0.76) | |
| 3-year duration  Patients remaining at risk, *n*  Event-free rate, % (95% CI) | 18  71.6 (59.0–84.3) | 245  92.0 (89.3–94.8) | 125  88.0 (83.6–92.4) | 112  93.4 (89.6–97.2) | 185  90.3 (86.9–93.6) | 70  87.4 (81.0–93.8) | 83  83.5  (77.6–89.4) | 125  92.7  (88.9–96.5) |

^a^EFS events occurred in *n*=8 (luminal A), *n*=9 (luminal B), and *n*=8 (basal-like) patients.

Abbreviations: CI, confidence interval; EFS, event-free survival; HER2, human epidermal growth factor receptor 2; HER2-E, human epidermal growth factor receptor 2–enriched; HR, hazard ratio; IC, immune cell; IHC, immunohistochemistry; PAM50, Prediction Analysis of Microarray with the 50-gene classifier; PD-L1, programmed death-ligand 1; *PIK3CA*, phosphatidylinositol-4,5-bisphosphate 3-kinase catalytic subunit alpha.

**Fig. S1.** HER2 staining patterns in breast cancer.


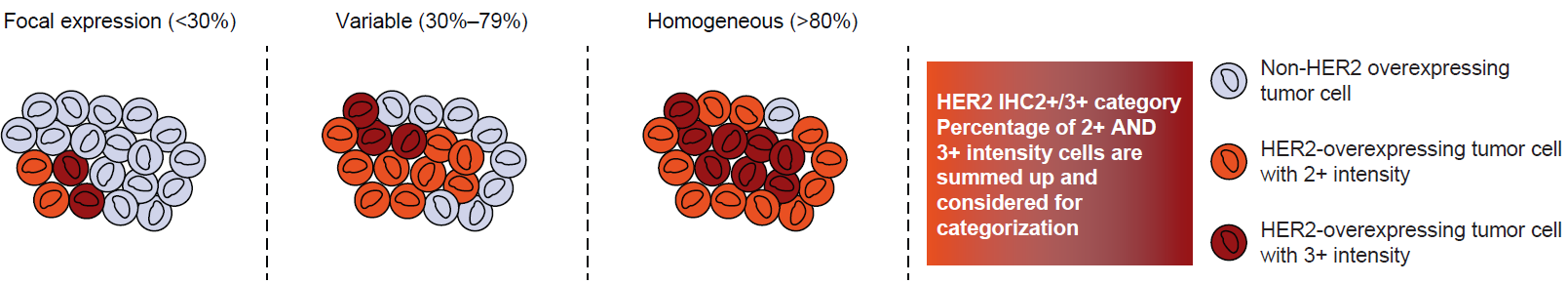


Abbreviations: HER2, human epidermal growth factor receptor 2; IHC, immunohistochemistry.

**Fig. S2.** Biomarker testing in KRISTINE (*N*=444).


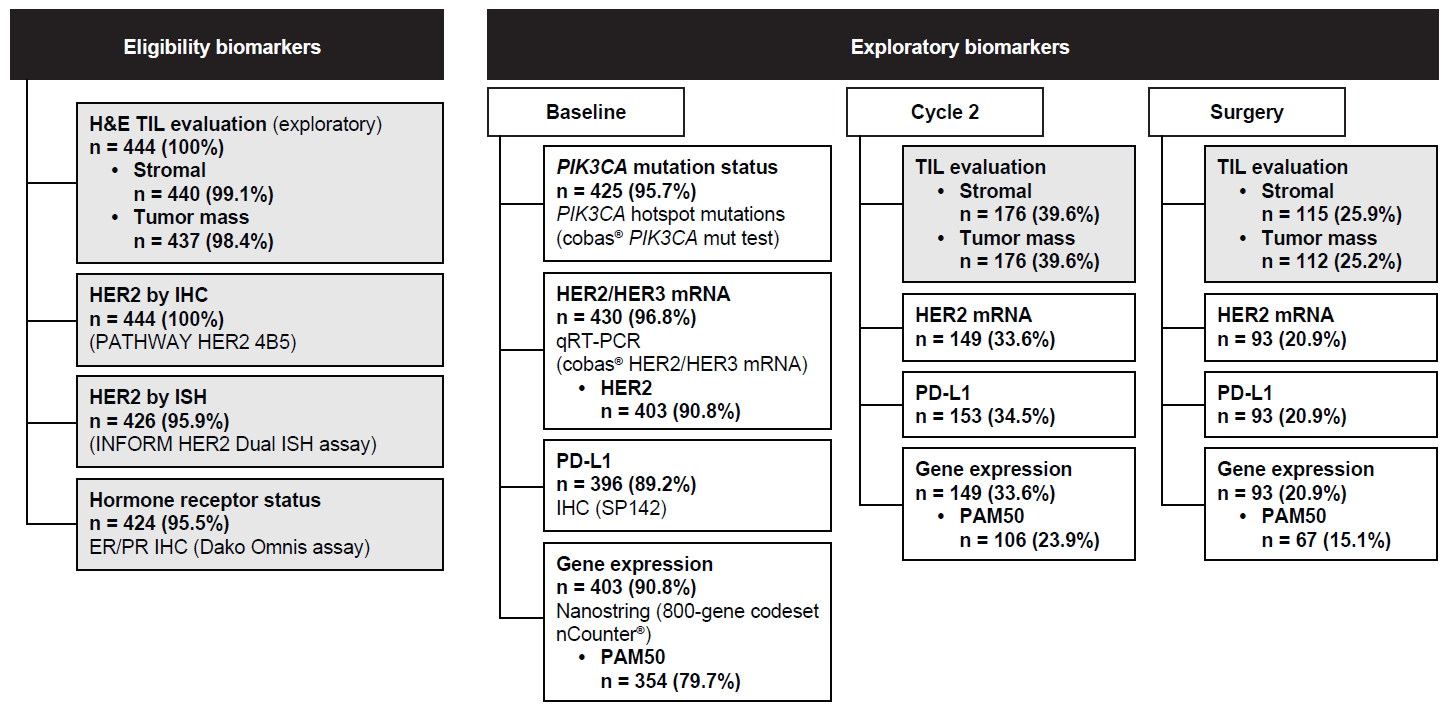


Abbreviations: ER, estrogen receptor; H&E, hematoxylin and eosin; HER, human epidermal growth factor receptor; IHC, immunohistochemistry; ISH, in situ hybridization; mRNA, messenger RNA; mut, mutation; PAM50, Prediction Analysis of Microarray with the 50-gene classifier; PD-L1, programmed death ligand 1; *PIK3CA*, phosphatidylinositol-4,5-bisphosphate 3-kinase catalytic subunit alpha; PR, progesterone receptor; qRT-PCR, reverse transcriptase polymerase chain reaction; TIL, tumor-infiltrating lymphocyte.

**Fig. S3.** Proportions of intrinsic subtypes among patients with known PAM50 subtype by **A,** the overall PAM50 population, and **B,** centrally determined hormone receptor status.^a,b^


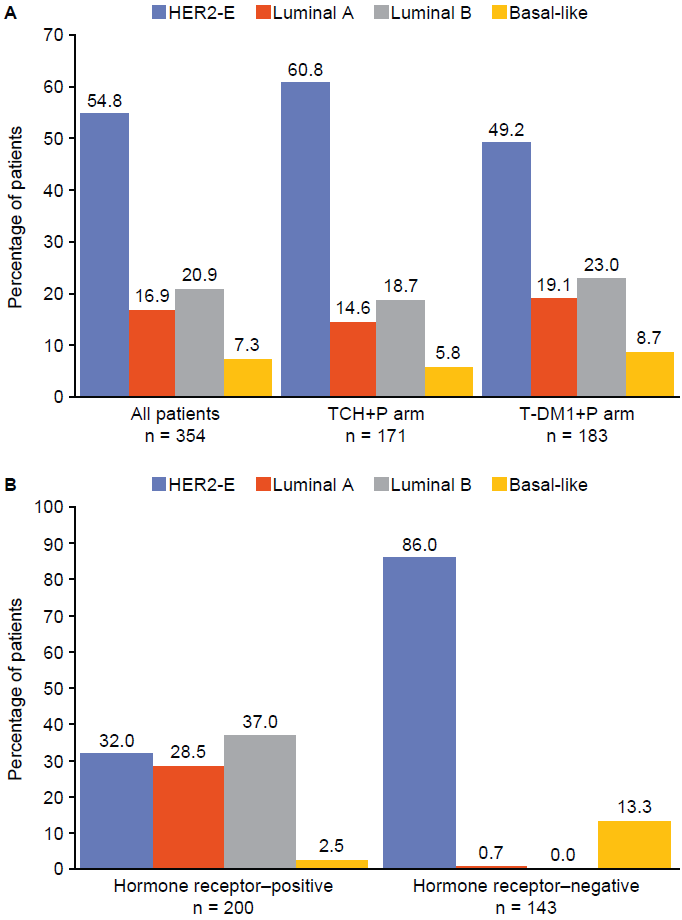


^a^Central hormone receptor status was unknown for 11 patients with known PAM50 subtype.

^b^No hormone receptor–negative patients had the luminal B subtype (0%).

Abbreviations: HER2-E, human epidermal growth factor receptor 2–enriched; PAM50, Prediction Analysis of Microarray 50; TCH+P, docetaxel, carboplatin, and trastuzumab plus pertuzumab; T-DM1+P, trastuzumab emtansine plus pertuzumab.

**Fig. S4.** Forest plot for PD-L1 status per hormone receptor subgroup.


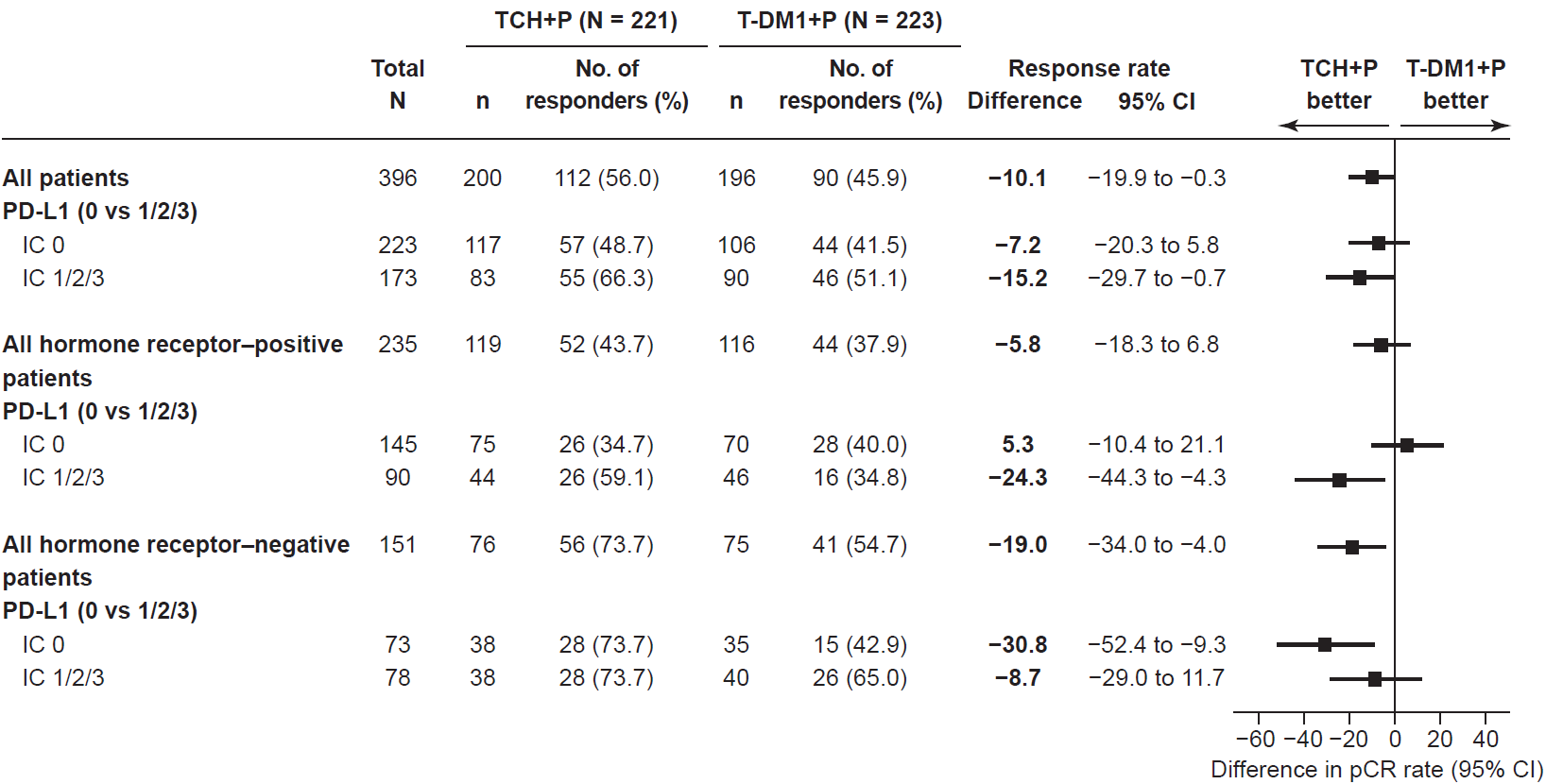


Abbreviations: CI, confidence interval; IC, immune cell; pCR, pathologic complete response; PD-L1, programmed death ligand 1; TCH+P, docetaxel, carboplatin, and trastuzumab plus pertuzumab; T-DM1+P, trastuzumab emtansine plus pertuzumab.

**Fig. S5.** pCR rates by immune genes and signatures.


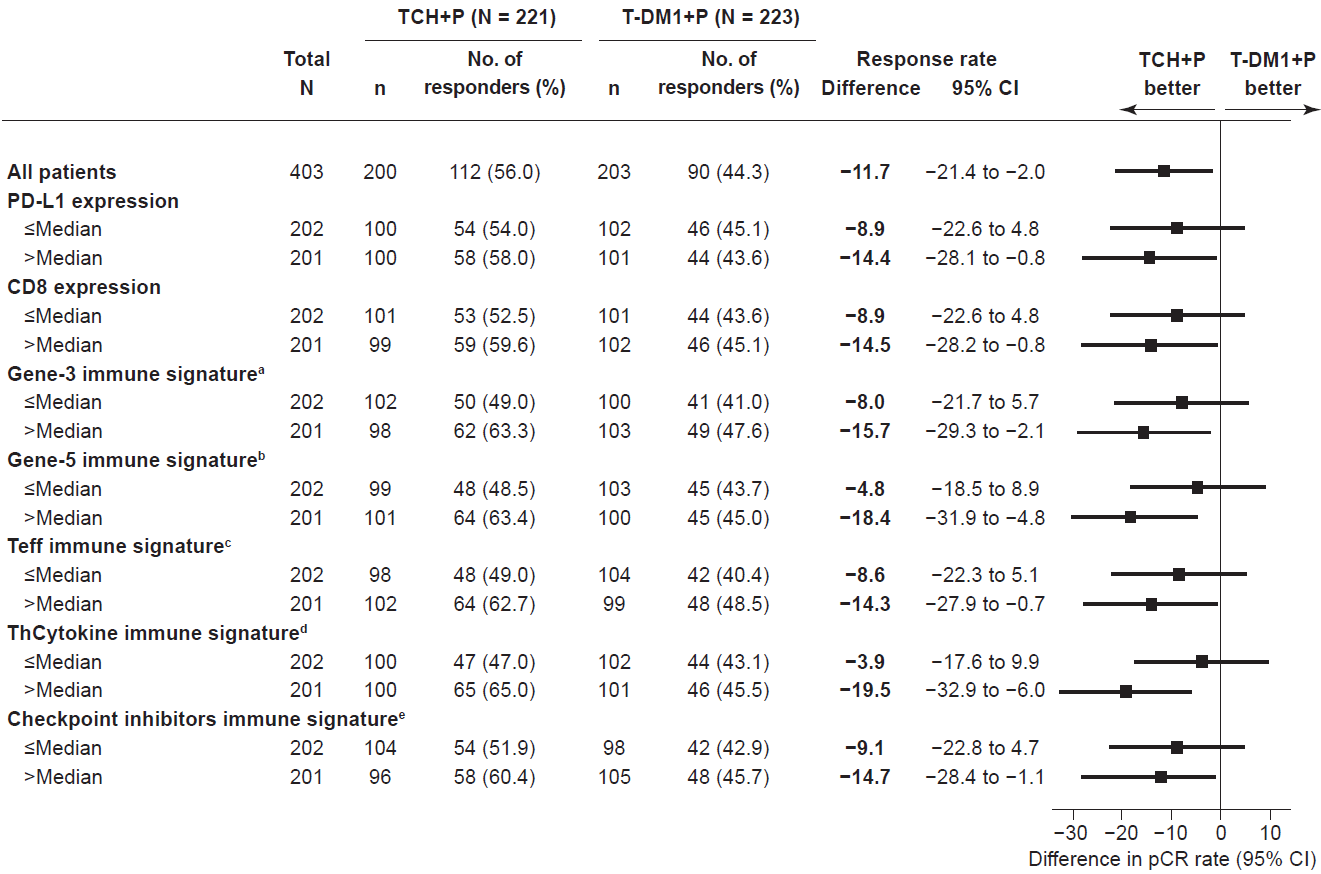


^a^3-gene: PD-L1/IFNG/CXCL9 >median vs. ≤median.

^b^5-gene: PD-L1/granzymeB/CD8/IFNG/CXCL9 >median vs. ≤median.

^c^Teff: CD8/granzymeA/granzymeB/perforin/IFNG >median vs. ≤median .

^d^ThCytokine: CXCL9/CXCL10/CXCL11 >median vs. ≤median.

^e^Checkpoint inhibitors: PD-L1/PD-L2/IDO >median vs. ≤median.

Abbreviations: CD, cluster of differentiation; CI, confidence interval; CXCL, C-X-C motif chemokine ligand; IDO, indoleamine 2,3-dioxygenase; IFNG, interferon gamma; pCR, pathologic complete response; PD-L, programmed death ligand; TCH+P, docetaxel, carboplatin, and trastuzumab plus pertuzumab; T-DM1+P, trastuzumab emtansine plus pertuzumab; Teff, T effector; ThCytokines, T-helper cytokines.

**Fig. S6.** Changes over time in **A,** PAM50 subtype; **B,** HER2 mRNA level; **C,** PD-L1 IC expression; **D,** dynamic PD-L1 IC expression with TCH+P treatment; **E,** dynamic PD-L1 IC expression with T-DM1+P treatment; and **F,** percentage of stromal TILs.


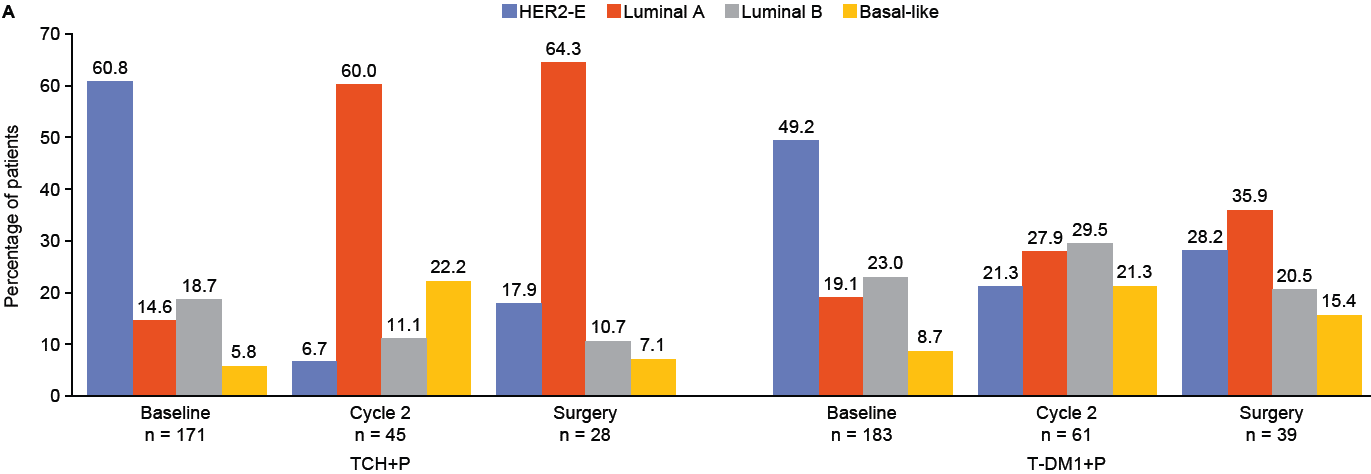


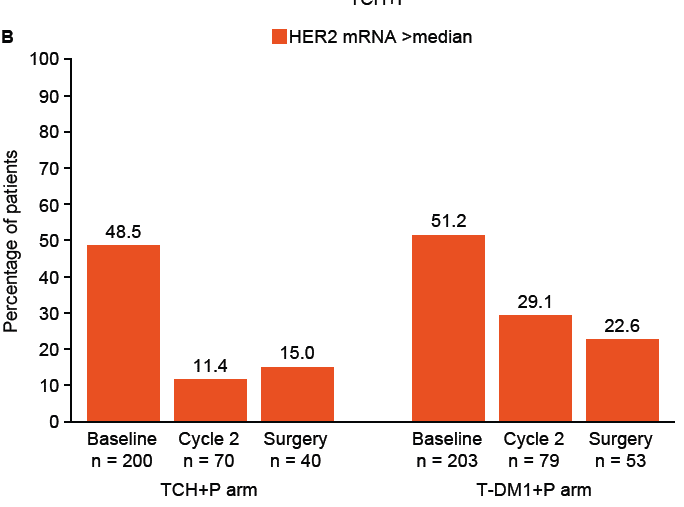


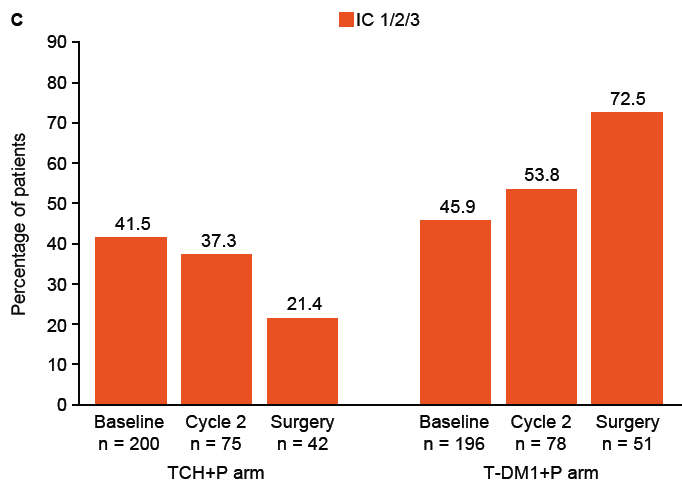


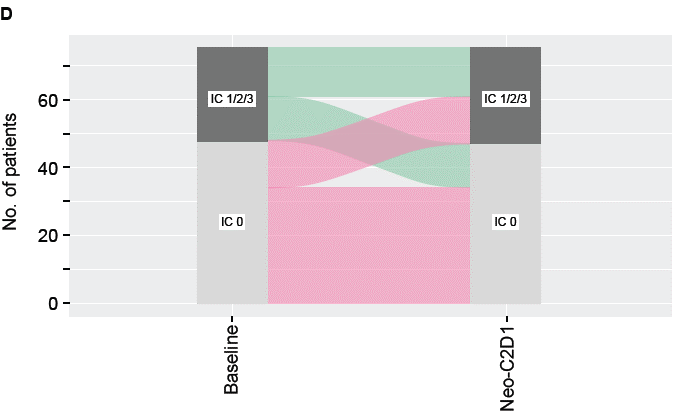


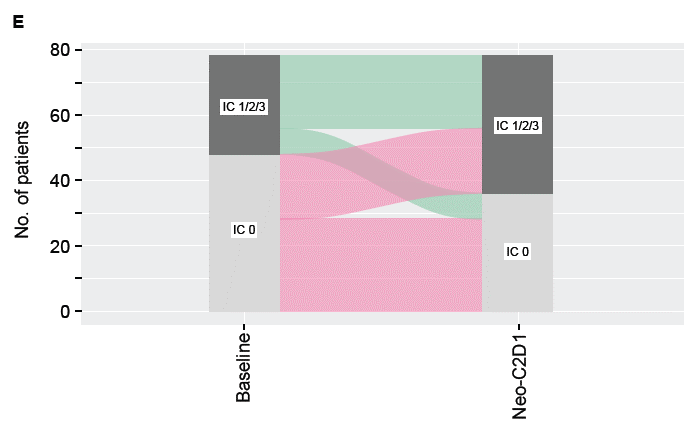


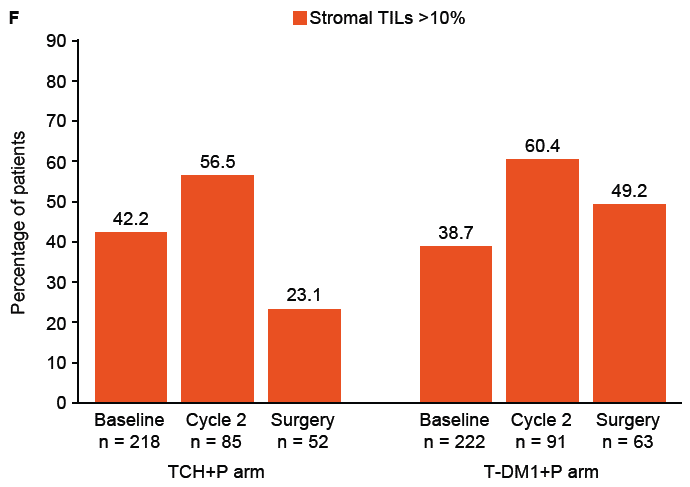


Abbreviations: C2D1, cycle 2 day 1; HER2, human epidermal growth factor receptor 2; HER2-E, human epidermal growth factor receptor 2–enriched; IC, immune cell; mRNA, messenger RNA; PAM50, Prediction Analysis of Microarray with the 50-gene classifier; PD-L1, programmed death-ligand 1; TCH+P, docetaxel, carboplatin, and trastuzumab plus pertuzumab; T-DM1+P, trastuzumab emtansine plus pertuzumab; TILs, tumor-infiltrating lymphocytes.

**Fig. S7.** Boxplots for immune signature change from baseline to neoadjuvant cycle 2 (ITT population)

1. Three-gene immune expression


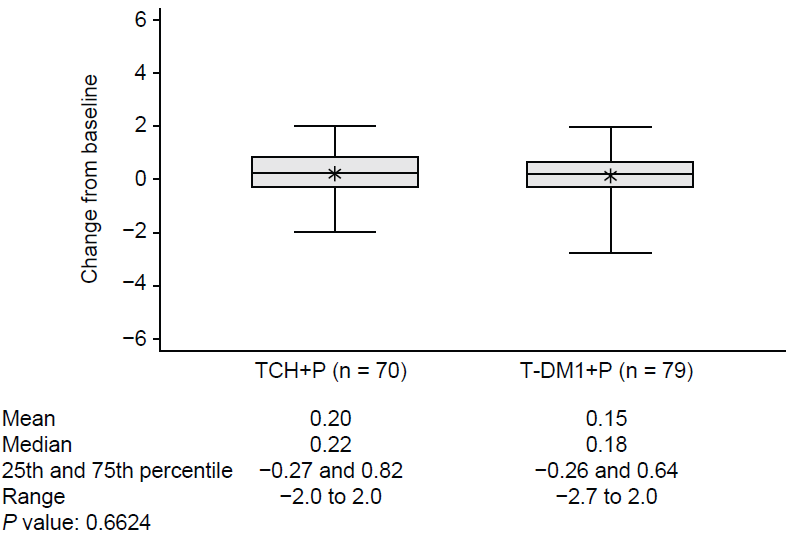


1. Immune signature ThCytokines expression


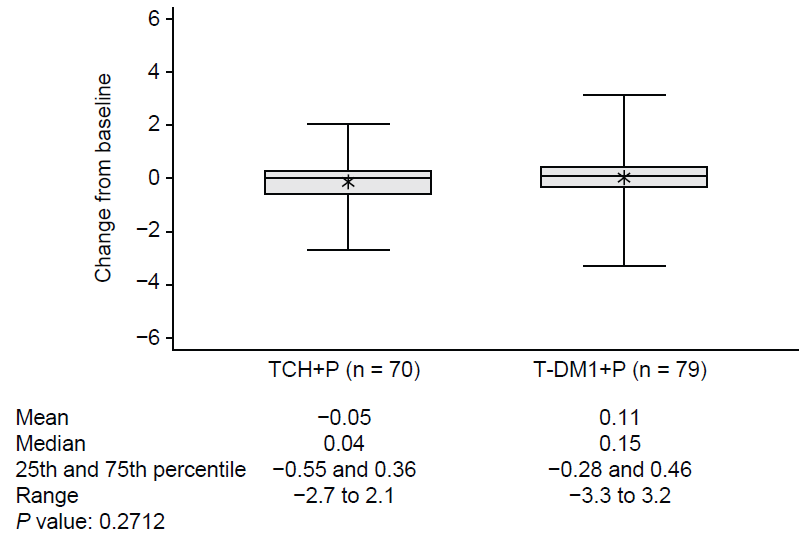


1. Five-gene immune expression


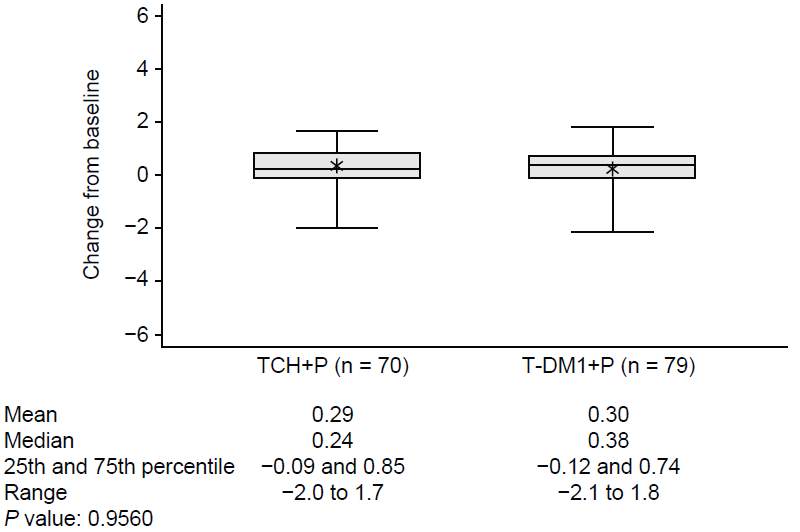


1. Immune signature checkpoint inhibitor expression


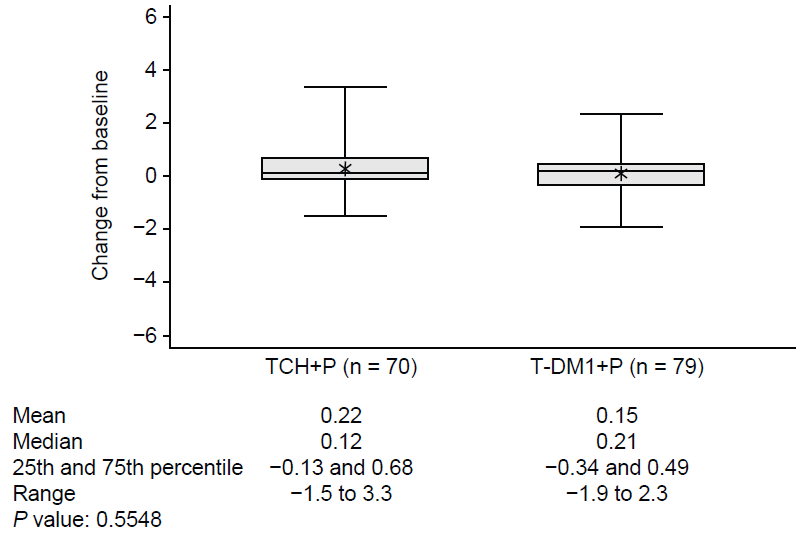


1. Immune signature Teff expression


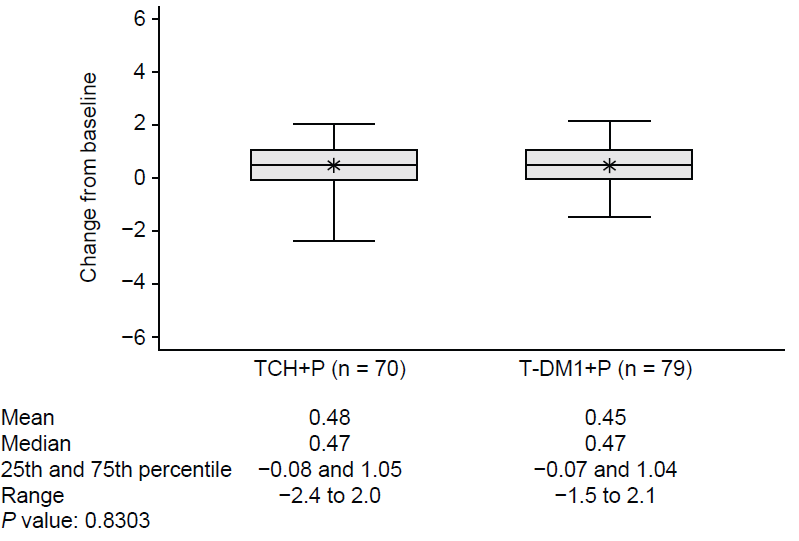


1. PD-L1 expression by treatment arm


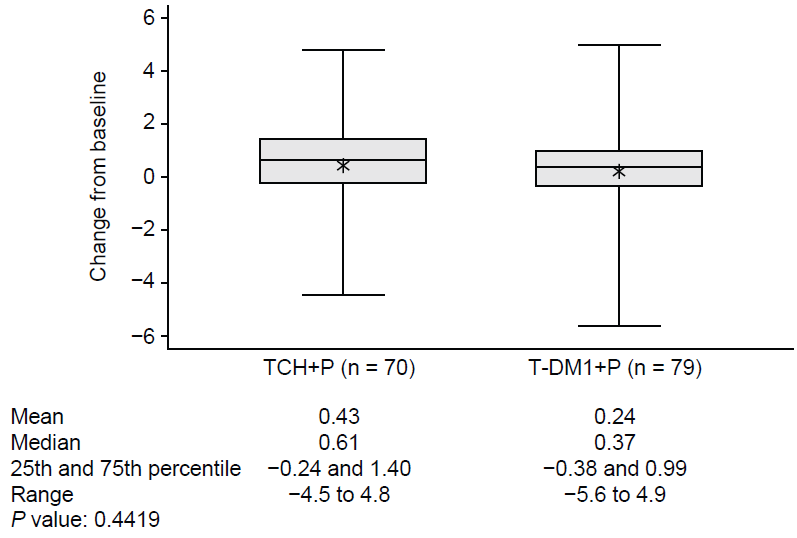


1. CD8 expression by treatment arm


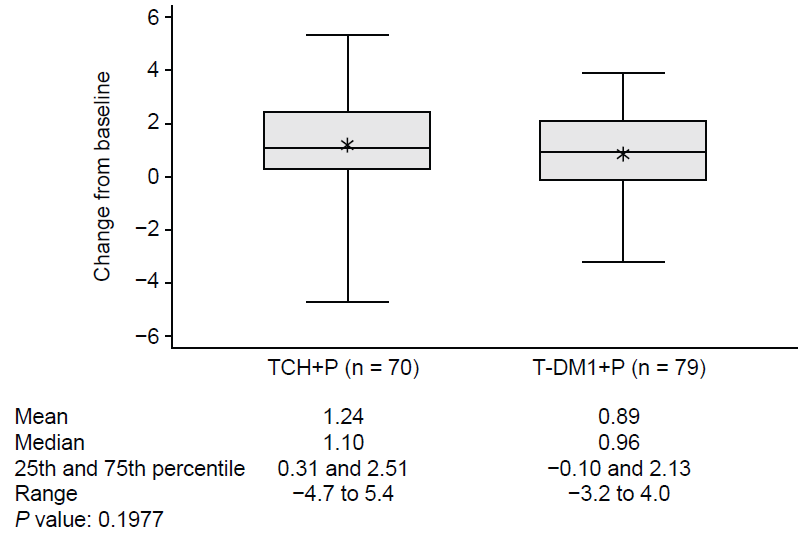


Abbreviations: CD8, cluster of differentiation 8; ITT, intent-to-treat; PD-L1, programmed death ligand 1; TCH+P, docetaxel, carboplatin, and trastuzumab plus pertuzumab; Teff, T effector; ThCytokines, T-helper cytokines; T-DM1+P, trastuzumab emtansine plus pertuzumab.

**Fig. S8.** Balloon plot of biomarker co-occurrence.


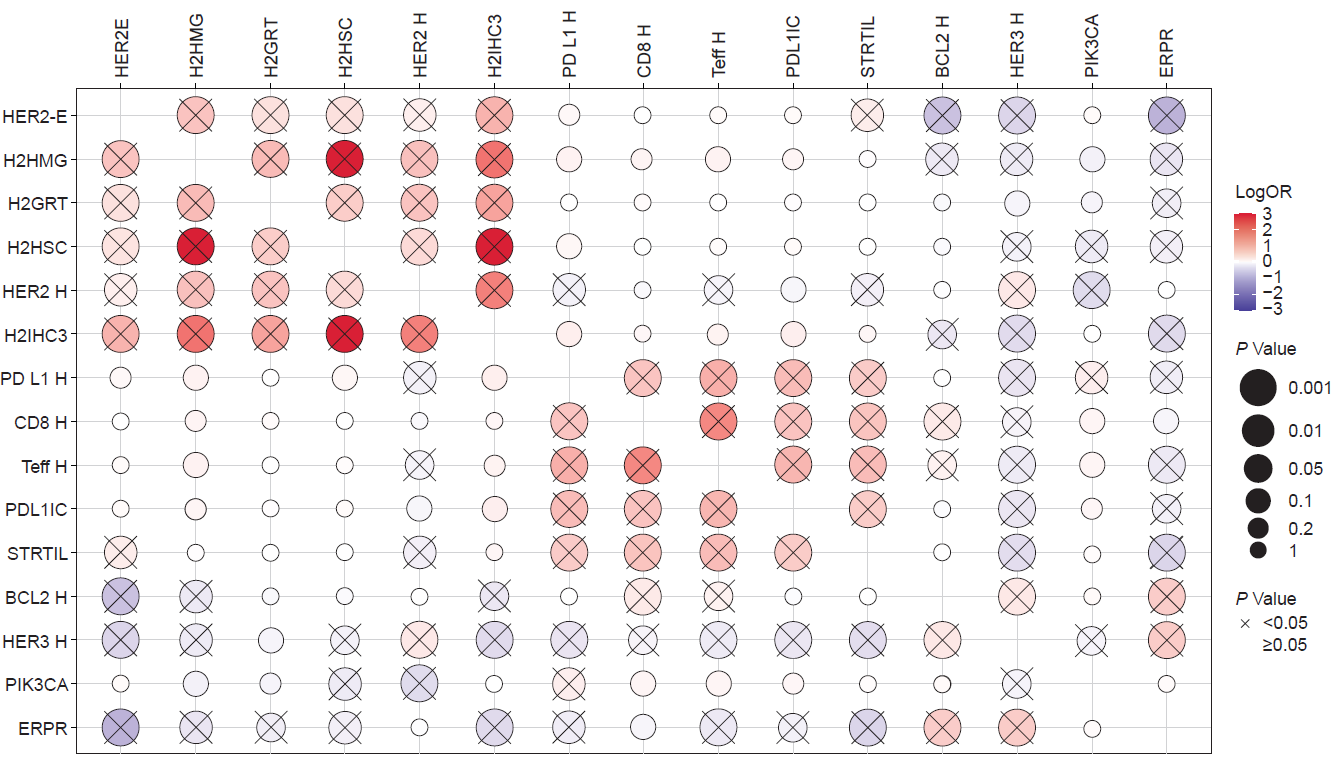


HER2E = HER2E PAM50 (vs. other).

H2HMG = HER2 homogeneous (vs. nonhomogeneous staining).

H2GRT = HER2 gene ratio (>4 vs. 2–4).

H2HSC = HER2 H score (>median vs. ≤median).

HER2 H = HER2 RNA high (>median vs. ≤median).

H2IHC3 = HER2 IHC3+ (vs. HER2 IHC 1+/IHC 2+).

PD L1 H = PD-L1 gene RNA high (>median vs. ≤median).

CD8 H = CD8 RNA high (>median vs. ≤median).

Teff H = Teff RNA high (>median vs. ≤median).

PDL1IC = PD-L1+ (IC 1/2/3 vs. IC 0).

STRTIL = stromal TILs high (11%–100% vs. 0%–10%).

BCL2 H = BCL2 RNA high (>median vs. ≤median).

HER3 H = HER3 RNA high (>median vs. ≤median).

PIK3CA = *PIK3CA* mutated (vs. nonmutated).

ERPR = hormone receptor positive (vs. hormone receptor negative).

Abbreviations: BCL2, B-cell lymphoma 2; CD8, cluster of differentiation 8; HER, human epidermal growth factor receptor; HER2-E, human epidermal growth factor receptor 2–enriched; IC, immune cell; IHC, immunohistochemistry; OR, odds ratio; PAM50, Prediction Analysis of Microarray with the 50-gene classifier; PD-L1, programmed death ligand 1; *PIK3CA*, phosphatidylinositol-4,5-bisphosphate 3-kinase catalytic subunit alpha; Teff, T effector; TIL, tumor-infiltrating lymphocyte.
